# Agrohydrological analysis of waterlogging impacts and mitigation in Ethiopia

**DOI:** 10.1038/s44264-026-00179-0

**Published:** 2026-09-02

**Authors:** Mosisa Tujuba Wakjira, Peter Molnar, Ruud Bartholomeus, Teklu Erkossa, Katrien Descheemaeker

**Affiliations:** 1 Plant Production Systems Group, Wageningen University & Research, Wageningen, The Netherlands; 2 Institute of Environmental Engineering, ETH Zurich, Zurich, Switzerland; 3 KWR Water Research Institute, Nieuwegein, The Netherlands; 4 Soil Physics and Land Management Group, Wageningen University & Research, Wageningen, The Netherlands; 5 Deutsche Gesellschaft für Internationale Zusammenarbeit (GIZ), Addis Ababa, Ethiopia

**Keywords:** Climate sciences, Ecology, Ecology, Environmental sciences, Plant sciences

## Abstract

Waterlogging is a major yet understudied constraint to cropland productivity. Here, we apply an agrohydrological modeling framework to assess the climatology of waterlogging (1981–2010), its yield impacts on major grain crops, and the potential for mitigation at a 500m x 500m grid resolution across Ethiopia’s rainfed agriculture (RFA) region. We estimate that areas within 39% of the grid cells in the RFA region are prone to waterlogging. Chickpea is the most sensitive crop, with average yield losses from 34% under low-severity to 97% under extreme-severity conditions, followed by wheat, barley, and faba bean (18–92% of yield loss). In contrast, maize and sorghum are generally tolerant except under extreme conditions. Our results also show considerable potential to reduce losses through soil drainage and use of tolerant crops. For instance, improved drainage could increase the current wheat yield on average by 57% under low waterlogging conditions. In flat areas where drainage is infeasible, productivity could be enhanced through using tolerant native crops and the expansion of rice production.

## Introduction

Waterlogging, a condition under which soil macropores are partially or fully filled with excess water, is a major constraint to crop productivity in more than 10% of global agricultural land^1,2^. In croplands, excess soil water results from intense rainfall over multiple days in rainfed systems or excessive field water application in irrigated systems. These conditions are often accompanied by prolonged field flooding (inundation), a rise in the shallow water table, or the emergence of a perched water table above impermeable soil layers such as claypans^3,4^. Both inundation and subsurface soil saturation are generally associated with the presence of clayey soils which have poor drainage characteristics. The intensity of waterlogging is also determined by topography, with low-lying, flat topographic positions highly susceptible to waterlogging^5^.

Waterlogging disrupts key processes in crop production. In the pre-planting stage, excess soil moisture reduces soil workability, thereby delaying seedbed preparation and undermining the desired soil structure during tillage^6^. In mechanized systems, it also restricts field trafficability, hindering tillage, planting, and crop management operations^7^. Such disruptions can shift the growth period and increase the risk of delayed emergence, heightened pest and disease pressure, and late-season water stress^8^. During crop growth, the primary impact of waterlogging arises from oxygen deficit in the root zone, which impairs root function and soil health^9^. Insufficient soil oxygen triggers anaerobic mineralization of organic matter, leading to CO_2_ and methane accumulation and root-zone toxicity^2,10^. Reduced oxygen circulation, together with toxic gas buildup, also inhibits nitrification and nitrogen fixation^11,12^, limiting nutrient and water uptake and suppressing photosynthesis. On average, photosynthetic rates in major food crops decline by about 40% under waterlogged conditions^13^.

The yield-reducing effects of waterlogging add an additional challenge to those posed by other weather and climate extremes, such as drought and heat stress, further constraining crop production. These impacts impede the efforts toward increasing food production for a growing global population and improving livelihoods in agriculture-dependent economies, particularly in developing countries^14^. Yet, waterlogging remains largely overlooked in current crop-climate impact studies that should inform practical decisions and adaptation strategies^1^. A recent commentary^15^ highlighted critical research gaps, including root morphological responses, interactions with biotic and abiotic stresses in the root zone, effects of varying waterlogging intensity in space and time, impacts on nutrient availability, crop tolerance, the effectiveness of management interventions, and the impacts of climate change on these various aspects. Here, we focus on three of these gaps, which are of vital importance in the context of rainfed agriculture (RFA) systems in Ethiopia: (i) assessing the spatial variability of waterlogging conditions, (ii) examining yield responses of the major cereals and legume crops, and (iii) evaluating the effectiveness of mitigation strategies.

**Fig. 1:**
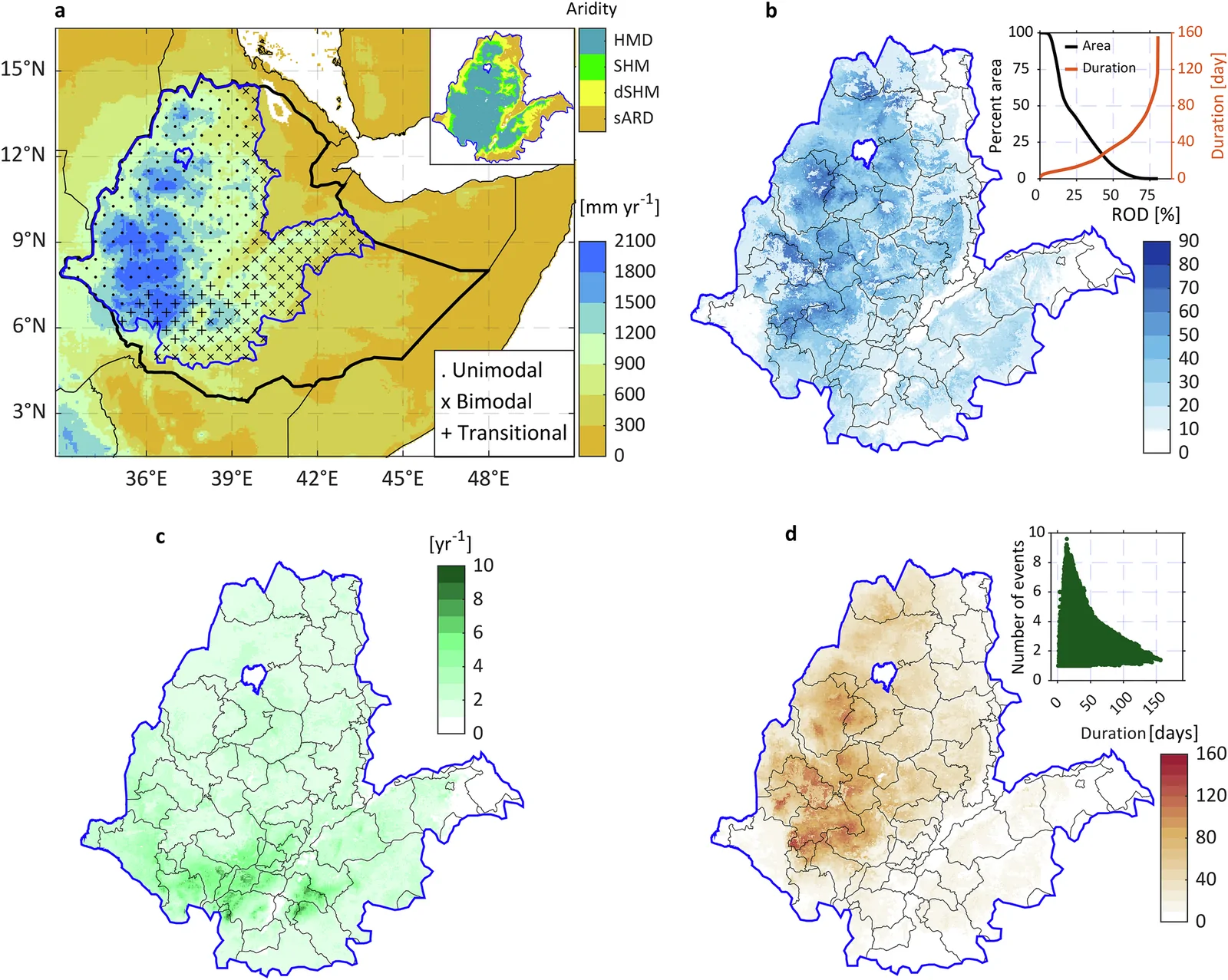
Climatology of rainfall and excess soil water conditions (1981–2010) across the rainfed agriculture (RFA) region of Ethiopia. **a** Mean annual rainfall and its temporal patterns (unimodal, transitional, and bimodal) based on CHIRPS rainfall^69^, with classifications following^20^. Inset: climate aridity classes of the RFA region: semi-arid (sARD), dry sub-humid (dSHM), sub-humid (SHM), and humid (HMD) following^106^. The RFA region boundary was defined following the ecoregion analysis map by^66^. **b** Climatological magnitude of root-zone oxygen deficit (ROD), expressed as percentage of air-filled soil porosity in the top 60 cm root-zone layer. Inset: cumulative percentage area-ROD (left axis) and duration-ROD (right axis) plots. **c** Average number of ROD events per year. **d** Average duration of ROD events. Inset: Scatterplot of the number of events versus the duration of events. Each data point represents a grid where ROD was observed.

Ethiopia provides a compelling case study because of socioeconomic and biophysical (climatic, edaphic, and topographic) reasons. Socioeconomically, the country is highly dependent on agriculture, dominated by smallholder rainfed systems that employ nearly 80% of the population and contribute more than one third of the national GDP^16^. Yet, productivity remains low, with substantial yield gaps in major crops^17^. Current agricultural and rural development strategies prioritize closing these yield gaps through sustainable intensification and diversification^18,19^. However, conditions that potentially exacerbate waterlogging are widespread across the agricultural region. Climatically, nearly half of Ethiopia’s rainfed croplands receive more than 1000 mm of rainfall annually (Fig. 1a), with 4–7 humid months per year^20^. The country has one of the largest vertisol extents in Africa^21^, covering about 18% of its landmass, mainly on flat slopes (<2%), where poor drainage exaccerbates waterlogging^22,23^. As a result, many cultivable areas remain underutilized or face yield reductions.

Past studies have assessed the yield reductions in annual crops and explored mitigation strategies, including surface drainage through broadbed-furrow (BBF) systems^24–28^, fertilizer optimization to offset nitrogen depletion caused by denitrification under waterlogging-induced anaerobic conditions^29–31^, cereal-legume intercropping to improve nitrogen uptake through root symbiosis^32^, and the selection of waterlogging-tolerant varieties and crop types^33–35^. However, these studies are limited to local-scale experiments, and a broader regional understanding in relation to the intensity and spatial extent, impacts on various crops, and potential for mitigation under different waterlogging severity conditions is lacking. Such insights are essential to inform the implementation of agricultural and rural development strategies.

To generate these insights, we conducted a spatially explicit analysis of rainfall-induced waterlogging impact and evaluated mitigation options across the rainfed agricultural region, using an agrohydrological modeling framework (see Fig. 7 and Methods) applied to the upper 60 cm of the soil root-zone depth at a 500 × 500 m grid resolution. In the framework, we followed four steps. First, we simulated soil water balance components: surface runoff, soil moisture, actual evapotranspiration, and deep percolation, accounting for soil and slope factors that influence the occurrence and intensity of waterlogging. Second, we identified waterlogging events using the simulated soil moisture content and quantified their magnitude, frequency, and duration. Third, we established a functional relationship between waterlogging intensity (WLI) and crop yield (the *yield response function*) to estimate yield losses. Finally, building on a previous evaluation of waterlogged soil management alternatives at locations across the RFA areas^23^, we assessed agronomic and drainage interventions for the mitigation of waterlogging-induced constraints on land productivity and yields across the RFA region.

The main relevance of this analysis lies in providing baseline information on the productivity constraints imposed by waterlogging for major crops in Ethiopia. The country’s current national food security and climate resilience strategy emphasizes increasing agricultural productivity, for which biophysical information at appropriate spatial scales is essential to support planning, implementation, and decision-making^36^. The information generated here can also serve as a reference for future assessments across different spatial scales (e.g., local contexts) and temporal domains, including climate-driven changes in waterlogging conditions and implications for crop yield. Beyond the practical relevance, the framework implemented in this analysis offers a flexible procedure for assessing waterlogging impacts that can be integrated with other crop and agroecosystem models for spatially explicit regional analyses. We demonstrate this by coupling the framework with the FAO water production function^37^ to estimate crop yield responses under moisture-excess conditions.

## Results

### Waterlogging conditions across the RFA region

Owing to the spatial and temporal distribution of rainfall (Fig. 1a), combined with the soil and slope characteristics, Ethiopia’s RFA region experiences varying conditions of excess soil water. Here, we present the climatology of these conditions based on our agrohydrological analysis for the period 1981–2010. During this period, nearly 10% of the RFA region experienced a Root-zone Oxygen Deficit (ROD) of 50% or higher. In other words, the soils in these regions were at least half-saturated (Fig. 1b). On average, these conditions persisted at least for ~ 35 days per event. High ROD events were evident predominantly in areas with mean annual rainfall exceeding about 1200 mm (Fig. 1a, b). In the main crop-producing humid region with unimodal and transitional rainfall regimes (see Fig. 1a), ROD lasted for up to three months, mainly from July through September (Supplementary Fig. 1). As expected, humid areas experienced continuous and prolonged waterlogging events, while most of the sub-humid areas exhibited relatively shorter and more intermittent waterlogging conditions (Fig. 1c,d). Intermittent ROD events were particularly common across the southern and southwestern parts of the RFA region with transitional (unimodal to bimodal) rainfall patterns, typically lasting fewer than 25 days per event. In drier areas, particularly dry sub-humid zones, ROD events were rare, weak in magnitude, and short in duration.

**Fig. 2:**
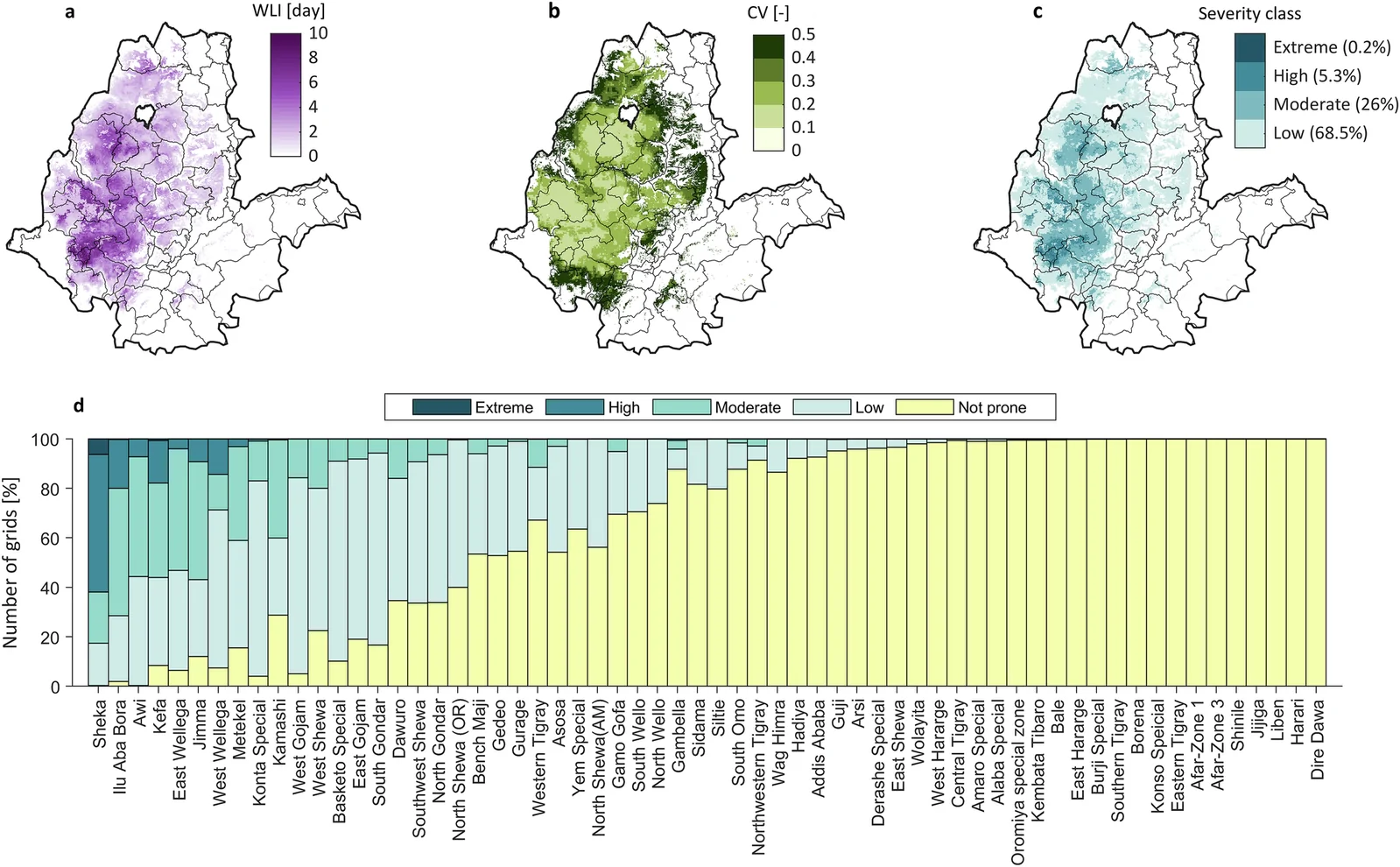
Waterlogging severity across Ethiopia’s RFA region. **a** Mean annual waterlogging intensity, WLI (1981-2010), expressed as the equivalent number of days per year with full saturation of the soil root-zone. **b** Interannual variability of WLI, shown as coefficient of variation (CV). **c** Spatial distribution of WLI by severity classes following^13^: low severity (*WLI* < 3 days), intermediate (3≤*WLI* < 6), high (6≤*WLI* < 9), and extreme (*WLI*≥9). Values in brackets show the percentage of waterlogging-prone areas within each severity class. **d** Extent of waterlogging severity classes within each administrative zone, expressed as a percentage of all grid cells in each zone. The administrative zones correspond to the pre-2010 administrative structure, for which the national crop yield data used in this analysis were reported^107^, and may therefore slightly differ from current administrative boundaries.

Waterlogging intensity (WLI), defined here as the combination of its magnitude and total annual duration, representing the equivalent number of days the soil remains saturated, is shown in Fig. 2a. Across the RFA region, 68.5 ± 5.5% (standard deviation *σ* in time over 30 years) of waterlogging-prone grids experienced low-severity waterlogging (*WLI* < 3 days), while 26.2 ± 3.4% experienced intermediate severity (3≤*WLI* < 6), mainly across the dry sub-humid, sub-humid, and parts of the humid areas during the climatological period 1981-2010 (Fig. 2c). High (6≤*WLI* < 9) and extreme (*WLI*≥9) waterlogging conditions occurred in humid areas of the west and southwest, accounting for 5.3 ± 2.4% of the waterlogging-prone area, with only 0.2 ± 0.5% (961 grid cells) experiencing extreme waterlogging conditions. Unlike areas with low-severity waterlogging, which showed high temporal variability (coefficient of variation, CV, up to 0.5), regions with moderate to extreme waterlogging exhibited low interannual variability (*CV* < 0.2), indicating persistent waterlogging limitations to crop production (Fig. 2b).

We also assessed the spatial extent of waterlogging severity within the administrative zones (Fig. 2d). Highly waterlogging-prone areas were found in Sheka, Kefa, Ilu Aba Bora, Awi, Wellega, Jimma, and parts of Metekel zones. In these zones, nearly 93% of grid cells, on average, experience waterlogging of low to extreme severity. In other zones, including major crop production corridors such as East and West Gojam, West and Southwest Shoa, North and South Gondar, Dawuro, North Shewa, and Gurage, low to moderate waterlogging was observed on average in 69% of grid cells in each zone, highlighting the widespread waterlogging conditions with potential impacts on crops.

### Crop yield losses in waterlogging-prone areas

**Fig. 3:**
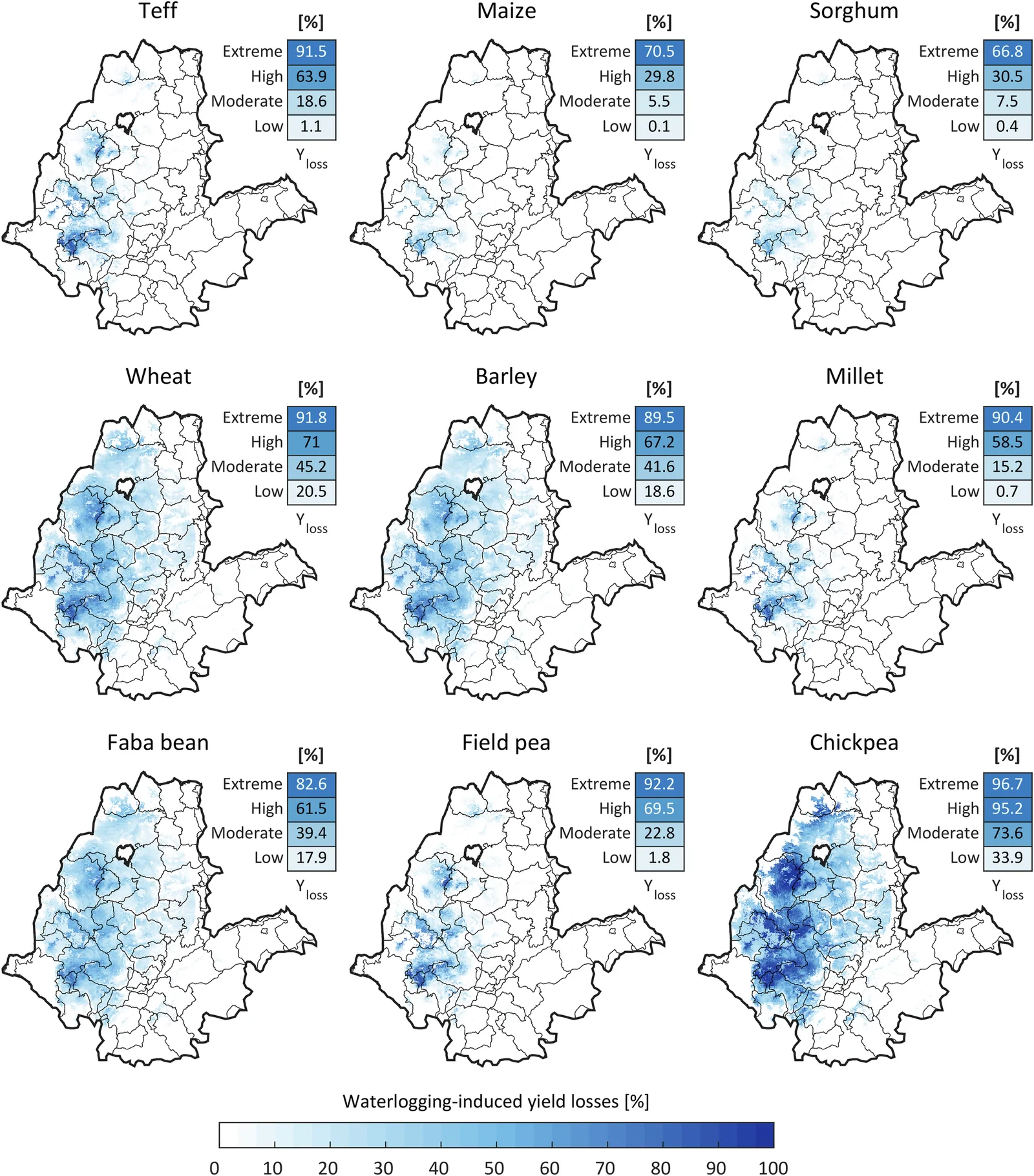
Yield losses under waterlogging conditions. Long-term (1981–2010) mean yield losses for cereal and legume crops under waterlogged conditions, expressed as a percentage of maximum yield obtained under optimal soil moisture conditions. Inset: area-averaged percent yield loss under the four waterlogging severity conditions.

To estimate waterlogging-induced relative yield losses, we used the *maximum yield* calculated under optimal soil moisture conditions using the yield response function (see Methods) as a reference. The effects of waterlogging on yields of major cereal and legume crops varied in space depending on the waterlogging severity and crop sensitivity (Fig. 3). Maize and sorghum showed relatively high tolerance to waterlogging compared to the other crops analyzed, with mean (± *σ* also in time) yield losses of 5.5 ± 0.5% for maize and 7.5 ± 0.5% for sorghum under moderate waterlogging severity, and none under low waterlogging severity. In fact, they benefited from WLI of up to 2.8 and 2.0 days (see Fig. 10). However, yield losses of these crops reached ~ 70 ± 3.5% and ~ 67 ± 3.4% under extreme severity, and ~ 30 ± 2.7% (both crops) under high severity conditions. Teff and millet showed similar responses, with threshold WLI of 1.8 and 2.1 days, respectively. In contrast, wheat and barley were highly sensitive, with wheat yield losses of ~ 90 ± 0.9%, 70 ± 2.1%, 43 ± 1.3%, and 20 ± 0.8% under extreme, high, moderate, and low waterlogging conditions, respectively (barley being slightly more tolerant). Faba bean losses were comparable to, but slightly lower than, those of wheat and barley, while chickpea was the most waterlogging-sensitive crop among the crops studied, with estimated yield reductions of 97 ± 0.01%, 95 ± 0.01%, 74 ± 2.2%, and 34 ± 1.4% across the four severity levels. Teff, millet, and field pea exhibited intermediate sensitivity, with average yield losses of 90–92%, 58–70%, 15–23%, and < 2% under extreme, high, moderate, and low intensity (see Fig. 3).

### Agronomic and soil drainage measures for waterlogging mitigation

**Fig. 4:**
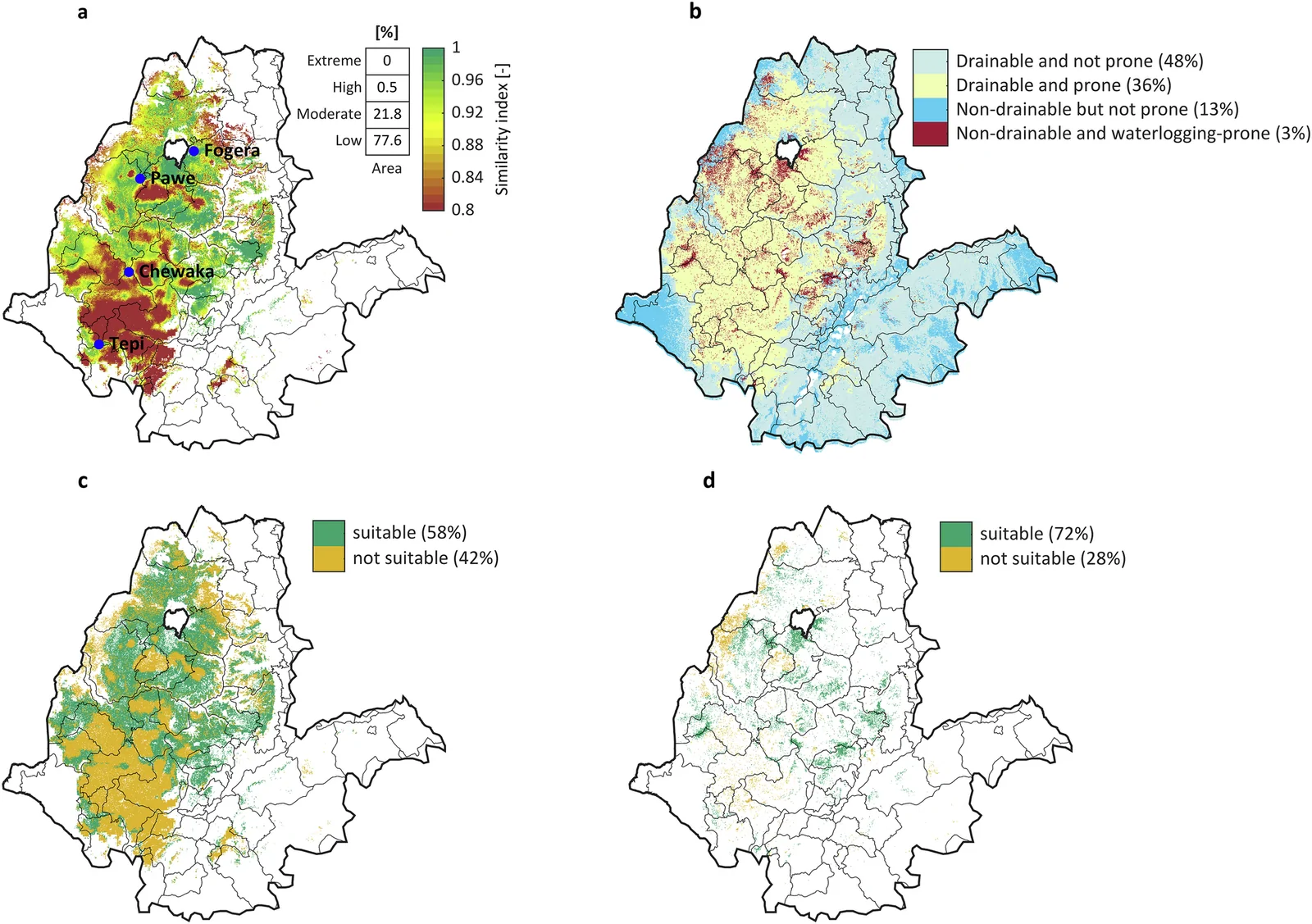
Agroecological suitability for rice cultivation in waterlogging-prone areas. **a** Grid-cell similarity to the reference rice-producing locations (Fogera, Pawe, Chewaka, and Tepi), based on climate, soil texture, and slope, derived using a biophysical analogs approach^41^. Inset: Percentage of areas (using a similarity threshold of 0.9) falling within each waterlogging severity class. **b** Distribution of waterlogging-prone areas by natural drainability, with slopes < 2% considered non-drainable. **c** Rice-suitable and unsuitable areas within drainable areas. **d** Rice-suitable and unsuitable areas within non-drainable areas.

The spatial extent of waterlogging-prone areas and associated yield losses across the RFA region highlights the need for effective adaptation and mitigation strategies to boost crop production. We evaluated two management interventions: agronomic and drainage measures. Agronomic practices, particularly the cultivation of tolerant crops, are the most viable solutions from both economic and environmental perspectives, avoiding the costs of drainage infrastructure as well as risks of erosion and nutrient leaching. In small-scale farming systems, which account for ~ 95% of crop production in Ethiopia, soil drainage measures are generally impracticable on flat fields (slope < 2%, ref. ^38^), because of the insufficient slope gradient. Such areas account for 3% of the RFA region, while drainable waterlogging-prone areas cover 36% (Fig. 4b). Rice cultivation is considered as a viable agronomic strategy in such contexts, due to its relatively high water demand and inherent tolerance to saturated soil conditions^39^. However, rice is still an emerging crop in Ethiopia with only about 65,000 hectares of land cultivated in 2019^19,40^, and experimental as well as practical experience across the diverse climates of the RFA region is still limited.

To identify areas that are potentially suitable for rice, we analyzed the agroecological similarity of individual grid cells to four major rice-producing reference sites, considering climate, soil texture, and slope, using the analogs method^41^. Approximately one-third of the RFA grids exhibited a similarity index ≥0.9 (that is, 90% or more similar to the reference sites), which we used as the threshold for potential suitability. Nearly all (99.5%) of these ’similar’ areas overlapped with areas under moderate (21.5%) or low (78%) waterlogging severity conditions (see Fig. 4a). Only 0.5% of the suitable areas were found within high severity, and none with extreme waterlogging. Accounting for landscape drainability, 58% of drainable and 72% of non-drainable waterlogging-prone areas were identified as suitable for rice cultivation (Fig. 4c,d). Unsuitable areas with low similarity to the reference locations, primarily differed in terms of climatic conditions. For example, waterlogging-prone areas in the northwestern part of the RFA region are drier than the reference locations, while severely waterlogged areas in the southwest are wetter. However, it should be noted that it is not entirely possible to conclude from this analysis alone whether these wetter areas are indeed unsuitable based solely on the four reference locations; thus, further investigation is required.

**Fig. 5:**
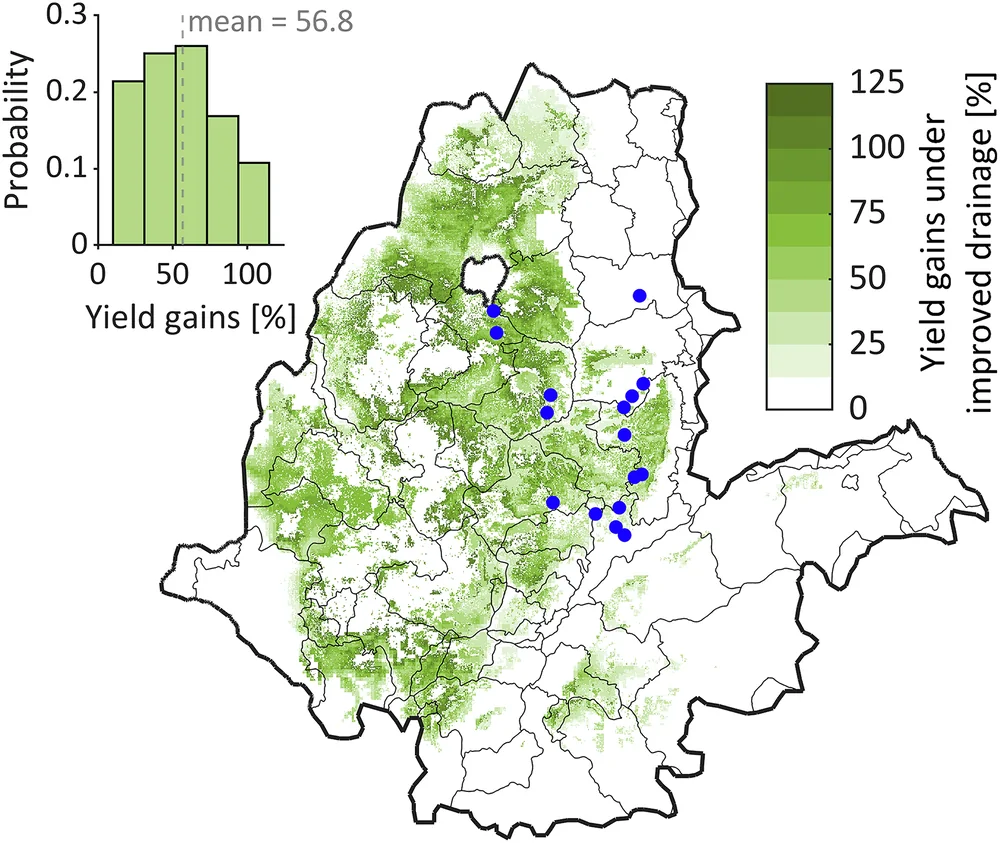
Wheat yield gains (%) through improved soil drainage in areas under low waterlogging severity. The map is based on a linear regression model of field trial data from 16 locations (blue dots), where yield responses to improved drainage were measured. Inset: probability distribution of yield gains across waterlogging-prone areas.

Soil drainage is a widely used strategy to manage excess soil water to improve land productivity in waterlogged areas^7^. In Ethiopia, it is largely small-scale, traditional and often ineffective, but improved practices, notably bed/ridge-and-furrow (BBF) seedbed preparation, have been tested in field trials at some locations across the RFA region^25,33^. We evaluated wheat yield gains from these improved surface drainage practices compared with traditional and/or no drainage practices, using the field trial data compiled from peer-reviewed and grey literature (Fig. 5 and Supplementary Table 3). The data suggest that for every one-day reduction in WLI through soil drainage, wheat yields increase by 37.6%. Therefore, in the current wheat-producing areas with low waterlogging severity, improved drainage can double the current actual yields, with a regional average gain of 56.8 ± 25.9% (*σ* in space). Because most trial sites are located in the major wheat-producing region (blue dots in Fig. 5), where waterlogging intensity (WLI) is generally low (≤3 days; Fig. 2) and climatological mean wheat yield loss due to waterlogging is up to 40% of the maximum yield (Fig. 3), it remains uncertain whether the observed linear relationship between improved drainage and yield gain holds under more severe waterlogging conditions. Consequently, a proper evaluation of the effectiveness of small-scale drainage practices across higher-severity waterlogging areas was not possible.

## Discussion

This analysis provides additional evidence from Ethiopia’s RFA region on the critical effects of waterlogging on crops and highlights the need to better account for waterlogging in future research and agricultural practices. The absence of adequate representation of waterlogging in many crop growth and agroecosystem models^1,15^ creates important information gaps for water management in humid agroecologies. For example, regional analyses often estimate crop yield responses to water under the assumption that crop transpiration and thus yield are primarily constrained by evaporative stress conditions, and yield responses under moisture-excess conditions are neglected^42,43^. A typical example is the widely applied FAO crop-water production (CWP) function, which links seasonal yield to water availability under water-limited conditions and assumes maximum yield when water is non-limiting^37^.

**Fig. 6:**
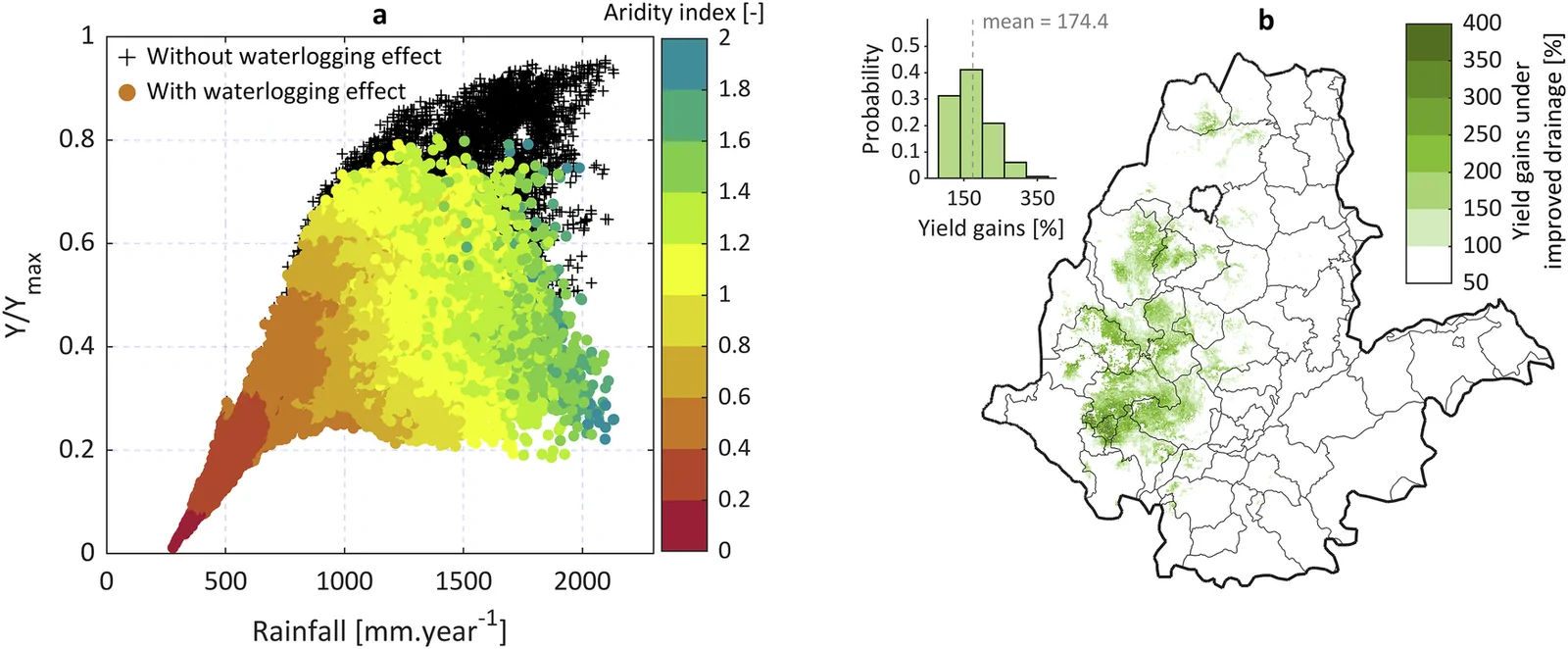
Wheat yield responses to water availability and yield gains through soil drainage. **a** Relative wheat yield ($Y/{Y}_{\max }$) under different climatic regimes as represented by mean annual rainfall. $Y/{Y}_{\max }$ without waterlogging effect was computed using the FAO crop water productivity function^37^: 1-*K*_*y*_(1-*ET*_*a*_/*ET*_*o*_), where *K*_*y*_ is crop-specific yield response factor obtained from^88^. $Y/{Y}_{\max }$ with waterlogging effect was determined by subtracting the estimated waterlogging-induced yield loss from the non-waterlogged yield. The color gradient of the dots represents the aridity index of each grid cell. Each point corresponds to a 5 × 5 km grid cell. Mean annual rainfall climatology (1981-2010) was obtained from CHIRPS. **b** Wheat yield gains achievable through improved drainage in areas under moderate to extreme waterlogging, assuming the linear regression model in Fig. 5. Inset: probability distribution of yield gains across waterlogging-prone areas.

Integrating crop and agroecosystem models with waterlogging impact models is therefore essential for representing the yield-reducing effects of excess water. In process-based models such as the Soil-Water-Atmosphere-Plant (SWAP) model^44,45^, the effects of waterlogging are represented through the Feddes root water uptake reduction function^46^ or through process-based parameterizations of crop oxygen stress^9^. In Fig. 6a, we illustrated waterlogging-induced yield reductions by coupling the FAO CWP function with the data-driven yield response curve implemented in this analysis. This approach extends the applicability of the CWP function, allowing it to estimate crop yield responses to seasonal moisture conditions not only under moisture-limited scenarios but also under moisture-excess conditions. For most crops, yield sensitivity under moisture-excess conditions in humid areas can be stronger than to water stress under moisture-limited conditions in dry areas (Supplementary Fig. 2), consistent with known general patterns of physiological responses of crops to oxygen deficit and associated stresses^47,48^. These responses also explain the low cropland suitability for cereals^49^ and legumes^42,50^ in the humid western and southwestern parts of the RFA regions, where yield losses from persistent waterlogging are most severe.

While crop yields in Ethiopia have increased in recent years due to improved inputs, including improved seed varieties and fertilizers, as well as better crop management, substantial attainable yield gaps still persist^51,52^. In waterlogged soils, the agronomic efficiency of these inputs and management practices is undermined^53^. The magnitude and spatial extent of the observed waterlogging impacts (Fig. 3) highlights the need for management interventions to help reduce the yield gaps. Nutrient use efficiency in waterlogged soils can be low because of enhanced nutrient leaching, disrupted nitrogen cycling, and restricted root nutrient uptake. This underscores the importance of proper seedbed preparation and drainage practices that minimize surface ponding and runoff during the growing season. Intervention measures, including soil drainage and seedbed preparation, need to be tailored to local conditions, accounting for waterlogging severity, natural drainability, and the crops affected, while integrating complementary strategies^23^.

In areas affected by low-severity waterlogging, which comprise most waterlogging-prone areas, predominantly in the major crop-producing areas, relatively small-scale and farmer-managed drainage practices, ranging from traditional ridge and furrow (RF) systems to improved broadbed and furrow (BBF) methods may substantially reduce yield losses. Effectiveness of these measures can be further improved by agronomic adjustments such as optimized planting dates, particularly for waterlogging-sensitive crops such as wheat, barley, faba bean and chickpea^54–56^. For example, Erkossa et al.^33^, through a four-year field experiment on vertisols in central Ethiopia, demonstrated that combining RF tillage with late planting can improve wheat yields by up to 60%.

In contrast, areas affected by moderate to extreme waterlogging likely require coordinated large-scale drainage interventions to reclaim affected potentially cultivable land^57^. Given the persistence of waterlogging impacts in these landscapes, improved drainage could substantially increase crop production and support regional food security. For example, if the 36.7% wheat yield gain observed per one-day reduction in WLI under low-severity waterlogging also holds under moderate- to extreme-severity, yields in these areas could increase on average by 174% (Fig. 6b). However, large-scale drainage plans must also account for ecological trade-offs, particularly impacts on wetlands and water-dependent ecosystem functions within agricultural landscapes^58^, especially when expanding cultivation into newly reclaimed lands.

In flat areas where drainage is not feasible, farmers may have to rely on alternative strategies such as cultivating tolerant crops. Our results suggest that native cereals such as maize, sorghum, and teff can serve as alternative crops under such conditions. In contrast, adaptation options for legumes are limited, as most legumes are highly sensitive to moderate to severe waterlogging. This limits cropping diversity in these areas, undermining the crucial role of legumes in crop rotation systems and as affordable protein sources. Breeding waterlogging-tolerant legume varieties could help address this gap^59^. Waterlogging tolerance of field pea (Fig. 3), and other legumes, such as grasspea^23^, under low-severity waterlogging helps maintain the rotation practices as well as enhanced livestock production.

Rice expansion into humid regions, where other grains are less suitable, is an additional alternative with the potential to enhance food security, farm incomes, and the national economy^60,61^. A comprehensive evaluation in Ethiopia^23^ found that rice cultivation on waterlogged soils generated substantially greater economic return than wheat grown under BBF-drained conditions, owing not only to grain yield but also straw production for livestock animal feed. Ethiopia’s National Rice Development Strategy 2020–2030^19^ targets expansion of rice from approximately 65,000 hectares in 2019 to 385,000 hectares by 2030 through both smallholder and commercial farming systems. Our findings provide complementary spatial information on areas where waterlogging constrains the suitability of traditional cereal and legume crops and where rice cultivation may offer a viable alternative to support this goal. Given that current rice cultivation is largely confined to areas with low to moderate waterlogging under predominantly smallholder systems, regions experiencing high to extreme waterlogging may represent potential future expansion areas, subject to detailed suitability assessments and environmental sustainability considerations.

While this study primarily examines the climatology of waterlogging and its yield-reducing impacts at high spatial resolution (500 × 500 m grids) to capture landscape-scale variability across the RFA region of Ethiopia, it places less emphasis on temporal dynamics. The primary focus is on identifying where waterlogging occurs, how it affects crops, and the extent to which these impacts can be managed. Two key aspects of temporal variability, however, are particularly important for effective adaptation and mitigation of these impacts and warrant further investigation. First, long-term trends in waterlogging, particularly those driven by climate change, are critical for informing future management strategies. However, future projections remain highly uncertain, highlighting the need for future research to reduce uncertainty and improve the robustness of predictions of rainfall and air temperature over East Africa. Moreover, while the current assessment relies on observed yield data, projecting waterlogging impacts under future climate conditions will require yield simulations using process-based crop models. Second, sub-seasonal variability strongly shapes the severity of impacts, as crop sensitivity to waterlogging depends on growth stage^62,63^. This variability directly influences farmers’ planting decisions as well as the design and capacity of drainage systems needed to mitigate risks^24,64^.

The modeling framework applied in this study, coupling a CN-based water balance model with crop yield response functions, relies on certain simplifications to address the complexity, data scarcity, and computational demands of the large geographical domain and high spatial resolution. Proper calibration of the CN-based model was not feasible due to the lack of spatially explicit data on surface runoff and soil moisture, a major constraint in Ethiopia. To enhance robustness, several adjustments were implemented, including CN scaling using observed runoff from 20 locations, slope-based CN adjustment, and consideration of soil clay and organic matter content. For site-specific applications, however, proper calibration with locally observed data is essential. Ongoing initiatives, such as the Coalition of the Willing^65^ that aims to alleviate such data scarcity in Ethiopia, will support future research.

One avenue for improving the framework for farm- and field-scale application is the inclusion of surface and subsurface lateral flow components. The present analysis is limited to rainfall-induced waterlogging, whereas in reality, particularly at the field scale, other hydrological processes, such as rises in shallow water tables and surface ponding due to runoff accumulation in downslope areas, can contribute to or exacerbate waterlogging. Such processes become especially important and are better represented at field scale. In addition, the yield response function used here is purely data-driven and does not capture sub-seasonal crop growth dynamics or stage-specific sensitivities. These aspects are more accurately represented by process-based crop models, which are well parameterized for such purposes^1^, and are strongly recommended for site-specific assessments to inform management decisions.

Despite the limitations outlined, the present findings can inform Ethiopia’s food security strategies at national and sub-national levels; however, more detailed, site-specific information is required to support local decision-making. As the country seeks to expand wheat self-sufficiency and implement its national rice development plan, tailoring adaptation and mitigation interventions to local waterlogging severity will be essential. Policies that support improvements in drainage technologies and supply chains, promote the breeding of waterlogging-tolerant legumes, and enable the expansion of rice into suitable humid regions can collectively strengthen resilience. Coordinated institutional planning that balances agricultural gains with ecological safeguards will be crucial for sustainably transforming waterlogged landscapes into productive, diversified, and climate-resilient farming systems.

## Methods

### Study area

The Ethiopian rainfed agricultural region (Fig. 1a) covers approximately 667,000 km^2^ of the country^66^, where crop production, integrated crop-livestock, and crop-livestock-agroforestry systems are the dominant economic activities^67^. The region is home to nearly 90% of the country’s population, with high population density in both rural and major urban areas^68^. The agroclimatic conditions range from semi-arid to humid, with annual rainfall ranging from 270 mm and 2100 mm across the region (Fig. 1a). The rainfall pattern has a unimodal distribution in the northern and northwestern parts, with the number of rainy months increasing toward the west; year-round rainfall in the southwestern part; and a bimodal pattern in the southern, southeastern, and eastern parts^20^. The main growing season, from May to September, accounts for nearly 88% of total grain production, while the shorter growing season, from February to May, contributes the remaining 12%, primarily across the southeastern half of the RFA region. Vertisols, leptosols, and nitisols are the three most abundant soil types across the RFA region, with substantial areas dominated by a high clay fraction^22^.

### Input datasets

**Fig. 7:**
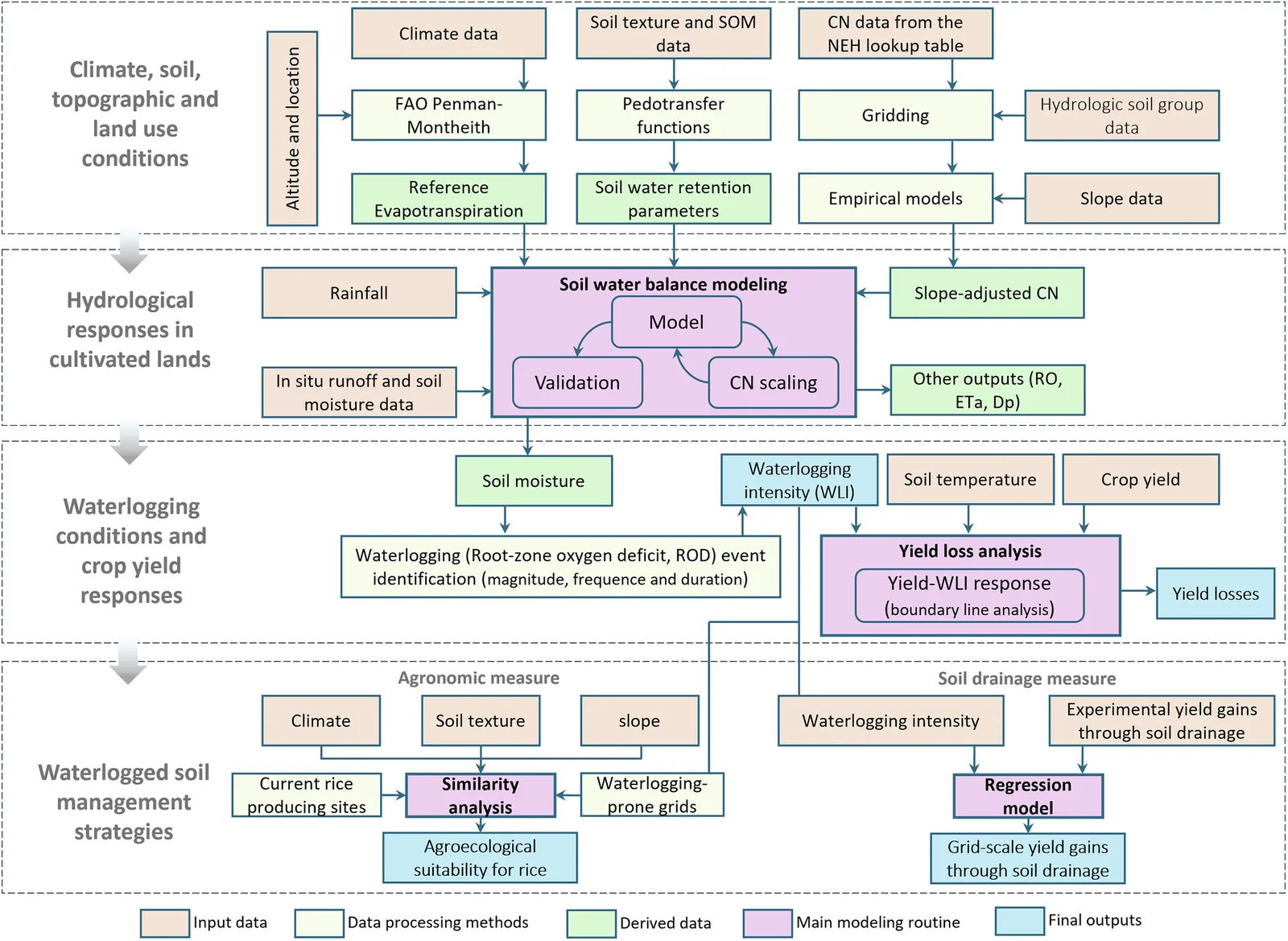
Agrohydrological analysis framework. CN: Curve Number, NEH: US Department of Agriculture National Engineering Handbook, ETa: actual evapotranspiration, RO: runoff, Dp: deep percolation.

Our analysis framework, including datasets, data processing, modeling steps is summarized in Fig. 7. The rainfall dataset used in the analysis was obtained from the Climate Hazards Infrared Precipitation with Station (CHIRPS 2.0)^69^ for the period 1981-2010. For air temperature, we used the Bias-Corrected ERA5 Land (BCE5) maximum and minimum temperatures for Ethiopia^70,71^. Both rainfall and temperature datasets are available at a ~ 5 km grid resolution (5 × 5 km). Other climate variables, including dew point temperature, solar radiation, and wind speed, were obtained from ERA5 Land^72^ and disaggregated from ~ 9 km to 5 km grid resolution using bilinear interpolation. Mean annual soil temperature data, used in the waterlogging impact analysis, was derived from ref. ^73^ at ~ 1 km resolution.

Soil texture and organic carbon content were derived from the SoilGrids database^74^ at ~ 250 m resolution. A 30 m digital elevation model used for the slope calculation was obtained from the Shuttle Radar Topography Mission (SRTM)^75^. Curve number (CN) values were obtained from the USDA lookup table^76^ and spatially mapped based on soil hydrologic group data developed by ref. ^77^. For calibration and evaluation of the root-zone water balance model, we compiled published runoff data from 40 locations and soil moisture data from 13 locations, drawn from both peer-reviewed and grey literature (see Supplementary Tables 1 and 2 in the Supplementary Information). The reported runoff and soil moisture data were either measured at experimental plots or, in the case of runoff, derived from locally calibrated model simulations across the RFA region. The crop yield data, used in the waterlogging impact modeling, was obtained for the years 2000, 2003–2007 and, 2010, from the annual agricultural sample survey reports by the Ethiopian Statistical Service^78^. For soil drainage performance evaluation, additional field trial data collected between 1981 and 2010, comparing traditional and improved drainage practices at 16 locations across the RFA region, were compiled from peer-reviewed and grey literature (Supplementary Table 3). For validation of predicted waterlogging-prone areas, we used data from 50 sites identified in the literature as waterlogged (including the 16 experimental sites) and 35 sites identified as non-waterlogged (primarily locations where soil and water conservation measures were implemented) (Supplementary Table 4).

The agrohydrological analysis was conducted at a 500 × 500 m grid resolution. However, to limit storage requirements and computational burden associated with high-resolution gridded daily climate data and to avoid additional uncertainties due to further spatial downscaling, climate variables were assumed to be uniform within each 5 × 5 km grid cell and were therefore maintained at this coarser resolution. Spatial variability in agrohydrological responses at the 500× 500 m scale was preserved by allowing non-climatic inputs, mainly soil and slope parameters, to vary at this finer resolution.

### Root-zone water balance modeling

The root-zone water balance in the top 60 cm soil layer was simulated using a conceptual agrohydrological modeling procedure without surface and lateral subsurface routing as implemented by ref. ^43^, with additional parameterizations to adapt the model for waterlogging simulation. The model simulates the change in soil moisture (*ΔSM* [mm]), surface runoff (*Q* [mm]), actual evapotranspiration (*ETa* [mm]), and deep percolation (*Dp* [mm]) at a daily time step (*t*), using rainfall (*P* [mm]) as input:

1 $$\Delta SM(t)=P(t)-Q(t)-E{T}_{a}(t)-{D}_{p}(t)$$

Surface runoff (*Q* [mm]) was estimated using the modified CN method^79,80^. In this modified model, the initial abstraction (*I*_*a*_ [mm]) is assumed to be 5% of the maximum surface retention (*S* [mm]), instead of 20% as in the original version. The main limitation of the CN method, aside from the assumption regarding *I*_*a*_, lies in its reliance on an empirical parameter (CN), which represents the effects of soil, land cover, and antecedent soil moisture conditions on the surface retention parameter *S*. However, its simplicity and computational efficiency, which are key requirements in our case, have contributed to its widespread use in many crop and agroecosystem models, including APSIM^81^, AquaCrop^82–84^, DSSAT^85^, and SWAT+^86^, among others. To address some limitations of the CN-based model, we adjusted the original CN values for terrain slope and antecedent moisture condition (AMC), and scaled them using observed runoff data from 20 locations across the RFA region. The adjustment of CN for *slope* [%] was performed outside the root-zone water balance modeling routine to compute a static adjusted CN (CN_*adj*_) (Eq.(3)) from the adjusted *S* (Eq.(2)), using the following empirical relationships^87^.

2 $${S}_{adj}=S\left(1.1-\frac{\,\mathrm{slope}}{\mathrm{slope}+{e}^{(3.7+0.02117\mathrm{slope})}}\right)$$

3 $$C{N}_{adj}=\frac{100}{\left(\frac{{S}_{adj}}{254}+1\right)}$$

**Fig. 8:**
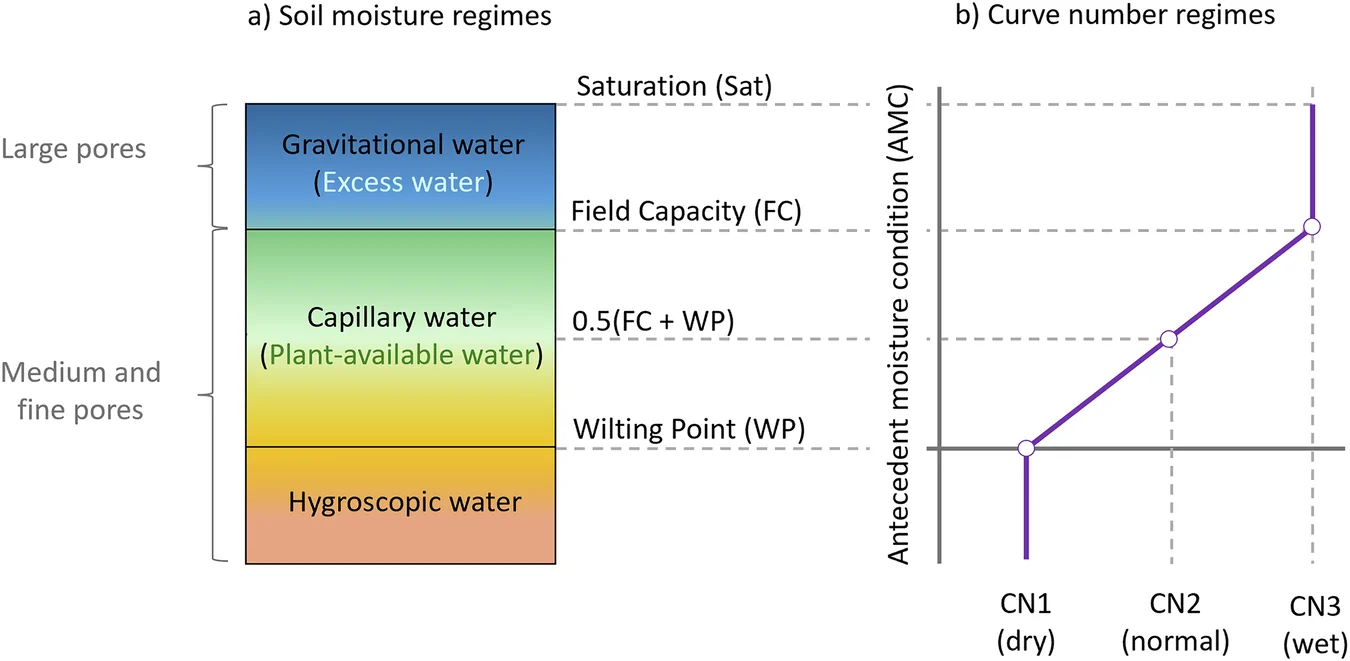
Conceptual representations of soil moisture and Curve number regimes. **a** Soil moisture constants and regimes. **b** Adjustment pattern for daily change in CN with antecedent moisture condition.

The adjustment of CN for AMC was made on a daily time step, considering the soil moisture regime as illustrated in Fig. 8. To this end, three CN regimes, namely CN1, CN2, and CN3, were used, corresponding to dry, normal, and wet AMCs, respectively. CN1 and CN3 were derived from CN_*adj*_, which represents CN2^87^.

4 $$CN1=C{N}_{adj}-\left(\frac{20(100-C{N}_{adj})}{100-C{N}_{adj}+{e}^{\left(2.533-0.0636(100-C{N}_{adj})\right)}}\right)$$

5 $$CN3=C{N}_{adj}\cdot {e}^{0.00673(100-C{N}_{adj})}$$

The daily curve number (*CN(t)*) was assigned as CN1 if antecedent soil moisture was below the wilting point (WP) and CN3 if it exceeded field capacity (FC). For AMC values between WP and FC, *CN(t)* was determined using linear interpolation, as illustrated in Fig. 8. The computed *CN(t)* was then adjusted using a scaling factor optimized by minimizing the root-mean-square deviation between simulated and observed runoff at 20 locations. Finally, the daily surface retention parameter *S(t)* was computed and used to estimate surface runoff *Q(t)* using the modified CN model^79,80^:

6 $$S(t)=254\left(\frac{100}{CN(t)}-1\right)$$

7 $$Q(t)=\left\{\begin{array}{ll}\frac{{(P(t)-0.071S(t))}^{2}}{(P(t)+1.349S(t))}, & P(t) > 0.071S(t);\beta > 2\\ \frac{\beta }{2}(\frac{{(P(t)-0.071S(t))}^{2}}{(P(t)+1.349S(t))}), & P(t) > 0.071S(t);\beta \le 2\\ 0, & \mathrm{otherwise}\end{array}\right.$$

Equation (7) accounts for slope-limited surface runoff in areas with very gentle to flat slopes^38^, by applying weighted adjustments to the simulated *Q* in grid cells with slopes below 2%^23^. The weighting factor was derived by normalizing the grid cell slope *β* relative to the threshold slope of 2%, such that a value of zero corresponds to flat areas and a value of one corresponds to the threshold slope. In other words, this adjustment is based on the assumption that, on flat fields, surface runoff ponds over the field and eventually infiltrates into the soil—an essential feature that must be represented in waterlogging assessments.

Actual evapotranspiration (*ET*_*a*_) was computed from the reference evapotranspiration (*ET*_*o*_), which was derived using the FAO Penman-Monteith equation^88^, and the soil moisture stress coefficient (*K*_*s*_), as follows:

8 $$E{T}_{a}(t)={K}_{s}(t)E{T_{o}}(t)$$

9 $${K}_{s}(t)=\left\{\begin{array}{ll}\frac{\theta (t)}{f({\theta }_{FC}-{\theta }_{WP})},\qquad\theta (t) < f({\theta }_{FC}-{\theta }_{WP})\\ 1,\qquad\qquad\qquad\,{\mathrm{otherwise}}\,\end{array}\right.$$

where *θ* [m^3^m^−3^] represents the volumetric moisture content. The parameter *f* is the critical moisture depletion factor that determines the readily available water, which is assumed to be 0.55, an average value for major cereal crops^88^. *θ*_*FC*_ and *θ*_*WP*_ denote the volumetric moisture content at field capacity (FC) and wilting point (WP), respectively, and were estimated using the pedotransfer function developed by ref. ^89^.

Soil moisture exceeding field capacity is considered gravitational water (Fig. 8a), which drains freely as deep percolation (*D*_*p*_ [mm]). *D*_*p*_ is computed as the difference between the volumetric water content at saturation (*θ*_Sat_) and at field capacity (*θ*_FC_), within a given root-zone depth (*Z*_*r*_ [m]), which is 60 cm in our case. However, in clayey soils, drainage is limited; therefore, *D*_*p*_ was set equal to the unsaturated hydraulic conductivity (*K*_*h*_). We estimated *K*_*h*_ [cm hr^−1^] as a function of soil moisture, following^90^:

10 $${K}_{h}=0.231{(\theta (t)-{\theta }_{r})}^{7.6512}$$

*θ*_*r*_ [m^3^ m^−3^] is residual moisture content, computed as a function of percent *clay* and organic carbon (*soc*), using the pedotransfer function proposed by ref. ^91^:

11 $${\theta }_{r}=0.00145+0.00253soc+0.00033clay$$

Deep percolation was therefore computed separately for clayey and non-clayey soils within the root zone (top 60 cm) using Eq. (12). The conversion factor 1440 accounts for the unit conversion from cm hr^−1^ to mm d^−1^ for *K*_*h*_, while the factor 1000 converts *Z*_*r*_ from meters to millimeters.

12 $${D}_{p}(t)=\left\{\begin{array}{ll}1440{K}_{h},\qquad\qquad\qquad\,\,\,{\mathrm{if}}\,{\mathrm{clayey}}\, ({\mathrm{i}}.{\mathrm{e}}., {\mathrm{clay}}\, > \,{40\% {\mathrm{and}}\, {\mathrm{sand}}}\, < \,{45\%)}\,\\ 1000{Z}_{r}(\theta (t)-{\theta }_{FC}),\qquad\,{\mathrm{otherwise}}\,\end{array}\right.$$

### Assessment of waterlogging conditions

We used root-zone oxygen deficit (ROD [%]) as a main variable in our assessment of the waterlogging conditions, their impacts, and mitigation measures. We defined ROD as the percentage of air-filled soil pore space occupied by water, calculated on a daily timescale *t* as:

13 $$ROD(t)=\left\{\begin{array}{ll}100\left(\frac{\theta (t)-{\theta }_{FC}}{{\theta }_{Sat}-{\theta }_{FC}}\right),\qquad\theta\,({\mathrm{t}})\, > {\theta }_{FC}\\0,\qquad\qquad\qquad\qquad\,{\mathrm{otherwise}}\,\end{array}\right.$$

Using the daily ROD values, we identified potentially impactful waterlogging events as periods of excess soil moisture lasting at least three consecutive days, because the impact on crops becomes significant for waterlogging of three days or longer duration^92^. For each year, over the study period from 1981 to 2010, we determined the total duration (*DUR* [day]) of impactful waterlogging and recorded the number of events. We then calculated the annual waterlogging intensity (*WLI* [day]) as the annual excess soil moisture corresponding to the mean annual oxygen deficit (*ROD*_*a*_) normalized by the root-zone depth (*Z*_r_ [mm]):

14 $$WLI=10\times RO{D}_{a}\times \left({\theta }_{Sat}-{\theta }_{FC}\right)\times DUR$$

Note that, *Z*_r_ is part of the waterlogging magnitude in Eq. (14), but it cancels out upon normalization of *WLI* with *Z*_r_ itself. In practical terms, WLI represents the equivalent number of days per year for which the soil remains saturated. For interpretation purposes, we classified waterlogging intensity as low (*WLI* < 3 days), moderate (3≤*WLI* < 6), high (6≤*WLI* < 9), and extreme (*WLI*≥9 days), following^13^.

### Model evaluation

**Fig. 9:**
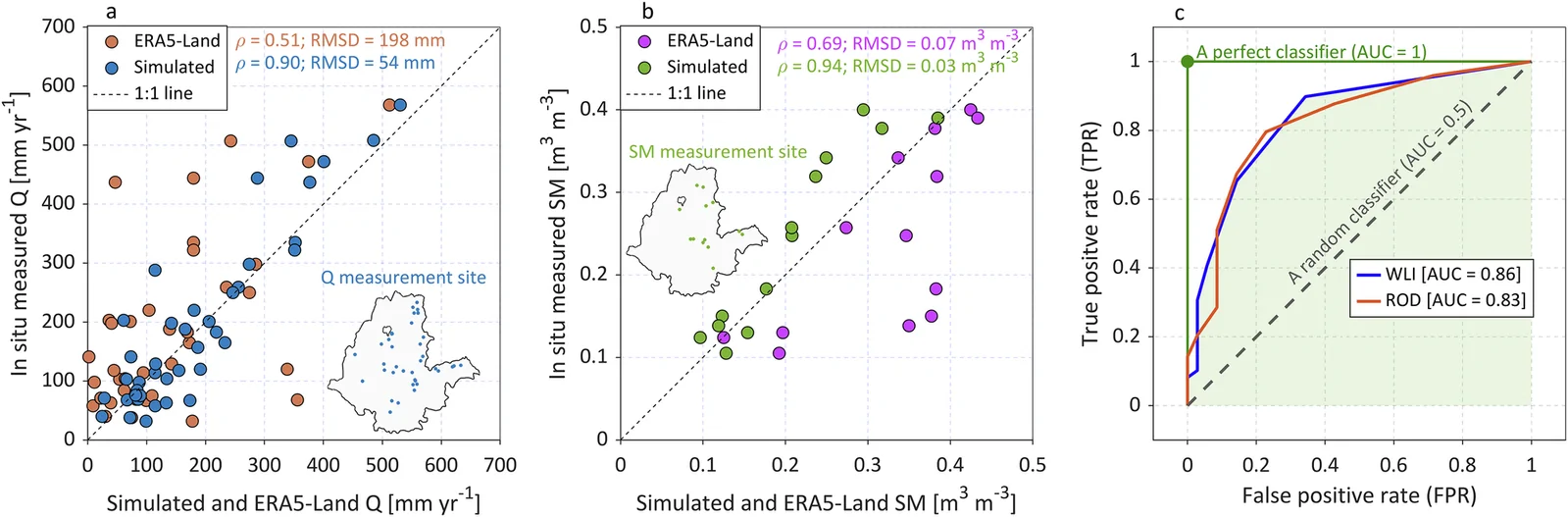
Model evaluation. **a** Comparison of simulated and ERA-Land reanalysis *Q* with in situ measurements at 40 locations shown in the inset map. The simulated and reanalysis values are grid values corresponding to the in situ measurement locations and years of measurement. **b** Same as in (**a**), but for *SM* at 13 locations. **c** Receiver Operating Characteristic (ROC) curve to evaluate the performance of simulated waterlogging intensity (WLI) and root-zone oxygen deficit (ROD) data for the prediction of the presence/absence of waterlogging based on 50 presence and 35 absence locations across the rainfed agricultural region.

Validation of the model through direct comparison between simulated and observed values was not possible due to limited observational data. Therefore, we evaluated model performance through indirect point-to-grid comparisons of simulated grid-cell values of *Q* and *SM* against in situ measurements reported in the literature at experimental locations across the RFA region (see Supplementary Table 1), using Pearson’s correlation coefficient (*ρ*) and root mean squared deviation (RMSD). Because in situ observational data are typically collected only at discrete locations, point-to-grid comparison remains one of the most practical and widely used approaches for evaluating spatially explicit model outputs and gridded datasets^93–96^. This method is based on the assumption that the gridded model estimates, if sufficiently accurate, are expected to capture information about corresponding point-scale observations^97^, as the same large-scale controlling processes, such as climatic conditions including rainfall and temperature, influence both. However, the agreement between point and grid values depends on spatial resolution and sub-grid heterogeneity in soil and topographic conditions^98,99^. Given the relatively fine spatial resolution of 500m × 500m in this analysis, scale mismatch is expected to be smaller compared to coarser-resolution applications. Accordingly, comparisons of simulated mean annual *Q* (Fig. 9a) and *SM* (Fig. 9b) with in situ observations yielded correlation coefficients of 0.90 and 0.94, with root mean squared deviations of 54 mm and 0.03 *m*^3^*m*^−3^, respectively. In addition, we compared the performance of the simulated *Q* and *SM* with that of independent global datasets derived from ERA5-Land, which provides relatively high spatial resolution and temporal continuity. Compared to the simulated data, ERA5-Land data showed weaker correlations and larger deviations from the in situ observations for both variables (see Fig. 9a,b) for both *Q* and *SM*.

We evaluate how well the *WLI* and *ROD* values derived from the simulated *SM* represent locations identified as waterlogged and non-waterlogged, based on 50 presence and 35 absence locations (see Supplementary Table 4), using Receiver Operating Characteristic (ROC) analysis^100^. ROC analysis assesses the trade-off between maximizing true positive and minimizing false positive predictions of presence. The ROC curve is constructed by plotting the true positive rate (TPR) against the false positive rate (FPR), which are defined as:

15 $$TPR=\frac{TP}{TP+FN}$$

16 $$FPR=\frac{FP}{FP+TN}$$

where *TP*, *FN*, *FP*, and *TN* are the numbers of true positive, false negative, false positive, and true negative predictions, respectively. The area under the curve (AUC) is used as the evaluation metric, with a value of 1 indicating perfect discrimination and 0.5 indicating random performance. The AUC values obtained for *WLI* and *ROD* were 0.85 and 0.83, respectively (Fig. 9c), suggesting that the simulated WLI and ROD reliably capture the presence and absence of waterlogging across the study area.

### Determination of yield responses to waterlogging

Crop response to waterlogging is primarily governed by impacts on root and leaf functioning^3,13,101^. Plant-scale responses are typically assessed through field experiments and well-parameterized crop models^102^. In this analysis, we do not examine plant-level functional responses, rather, we focus on yield (crop-level) responses, assessing spatial variability in waterlogging conditions on a climatological timescale (considering the period 1981-2010), and the associated yield responses using yield data of nine cereal and legume crops grown in Ethiopia, namely, teff, maize, sorghum, wheat, barley, millet, faba bean, field peas, and chickpea. We estimated crop yield responses to waterlogging by establishing a functional relationship between yield and waterlogging intensity (WLI), referred to as the yield response function, following the boundary line analysis framework of ref. ^49^. For this, we used yield data from the annual Agricultural Sample Survey (AgSS)^78^ for the main growing season over seven years (2000, 2003-2007, and 2010) from 62 administrative zones.

**Fig. 10:**
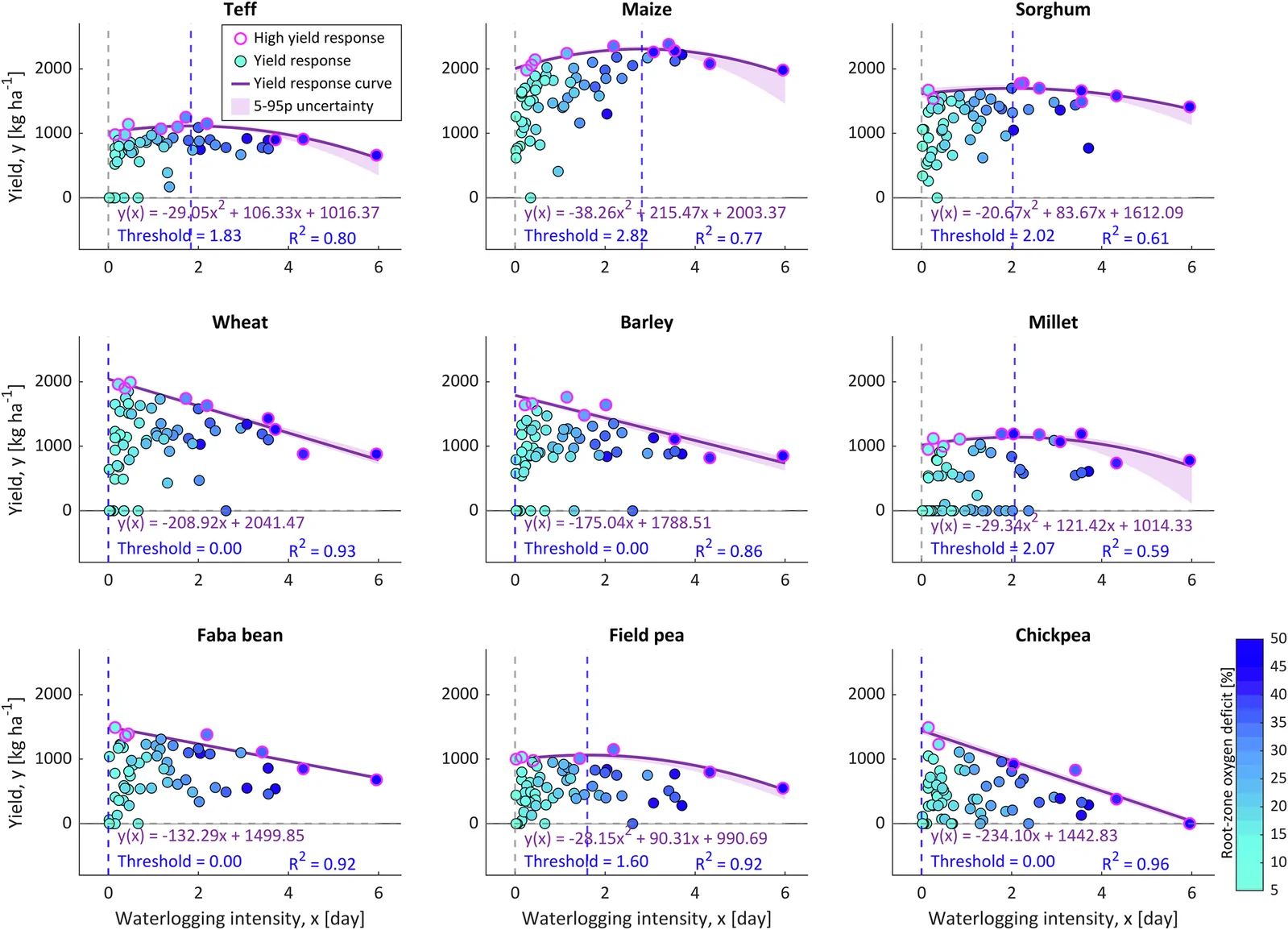
Yield responses of nine crops to mean annual waterlogging intensity (WLI) across the rainfed agricultural (RFA) region of Ethiopia. Each data point represents the seven-year mean (2000, 2003–2007 and, 2010) of zone-averaged yield for an administrative zone. The color gradient indicates average root-zone oxygen deficit. Pink circles represent the sampled high yield response (HYR) data used to define the model (purple line). The purple shade indicates the 5-95 percentile uncertainty band based on a leave-one-out sensitivity analysis. The blue dashed line marks the threshold WLI above which yield begins to decline.

To determine the yield response function for each crop, we first calculated the average WLI for each administrative zone for the years corresponding to the available yield data. We then plotted the zone-averaged yield of individual crops against the corresponding WLI values to visualize the response pattern, as illustrated in Fig. 10. From these scatterplots, we sampled data points along the upper envelopes using the convex hull algorithm^103^. These selected points, referred to as *high yield response* (HYR) data (shown in pink in Fig. 10), represent locally achievable yields across the range of WLI values. The number of data points selected as HYR depends on the nature of the response pattern. To assess the sensitivity of the response model to individual HYR data points, we performed a leave-one-out sensitivity analysis^104^ and illustrated the resulting 5^*th*^-95^*th*^ percentile range of the model with the purple shading in Fig. 10.

To model crop response, we fitted polynomial functions to the HYR data points for each crop. For wheat, barley, faba bean, and chickpea, the yield response to WLI was linear, indicating a consistent decline in yield with increasing waterlogging intensity. In contrast, the responses of the remaining crops were best represented by second-degree polynomials. For these crops, we identified a threshold WLI (marked by the blue dashed line in Fig. 10) beyond which waterlogging has a negative effect on yield. The resulting models are indicated for each crop also in Fig. 10. We then applied these models at hyper-resolution (500 × 500 m) grid-scale to simulate yield losses.

Yield loss under waterlogged soil conditions can vary depending on soil temperature and the depth at which waterlogging occurs^9^. In our impact modeling, we accounted for the effects of soil temperature (*T*_*soil*_) in addition to soil type, which we described in the framework above. However, we did not account for soil depth, as our analysis considers only the top 60 cm soil layer, which does not necessarily capture the full root-zone depth of individual crops. To quantify the effect of *T*_*soil*_ [°C], we followed a similar approach to that used for the yield-WLI model to construct a yield-*T*_*soil*_ model (Supplementary Fig. 4) and applied it at the grid scale. Finally, yield loss (*Y*_*loss*_ [%]) under waterlogged conditions was expressed as a percentage of the maximum yield, defined as the yield corresponding to the threshold WLI:

17 $${Y}_{loss}=\left\{\begin{array}{ll}100\times {K}_{T}\left(1-\frac{Y(WLI)}{{Y}_{max}}\right),\quad{T}_{soil} > {T}_{thr}\\{100}\left(1-\frac{Y(WLI)}{{Y}_{max}}\right),\qquad\qquad\,{\mathrm{otherwise}}\,\end{array}\right.$$

where *Y*(*WLI*) is yield response to WLI derived from the yield-WLI model, *Y*_*max*_ is *Y*(*WLI*) corresponding to the threshold WLI, and *K*_*T*_ is soil temperature factor and is computed as:

18 $${K}_{T}=\left(2-\frac{Y({T}_{soil})}{{Y}_{max}}\right)$$

Equation (18) assumes that soil temperatures exceeding the threshold (*T*_*thr*_) elevate crop oxygen demand, thereby accelerating oxygen depletion and increasing the risk of crop damage^2,9^.

### Evaluation of management alternatives

To assess the agroecological suitability for rice expansion in waterlogging-prone areas across the broader RFA region, we conducted a similarity analysis using the analogs approach proposed by ref. ^41^. In this method, a dissimilarity index is calculated based on the Euclidean distance between relevant variables at a reference location and a location of interest. We selected four major rice-producing areas in Ethiopia, Fogera, Pawe, Chewaka, and Tepi, as reference sites (Fig. 4a). We computed the dissimilarity index (*I*_*diss*_) at each grid cell by comparing its climatic aridity, soil clay and silt content, and slope against the average conditions of the four reference sites, as follows:

19 $${I}_{diss}={\left(\sum {W}_{i}{({X}_{i}^{ref}-{X}_{i}^{grid})}^{2}\right)}^{1/2}$$

where ${X}_{i}^{\,\mathrm{ref}\,}$ and ${X}_{i}^{\,\mathrm{grid}\,}$ represent the values of agroecological variable *i* at the reference and grid locations, respectively, and *W*_*i*_ is the weight assigned to each variable. We assigned weights of 0.5, 0.2, and 0.3 to climate, soil, and slope, respectively, based on the coefficient of variation (across grid cells) of the predetermined dissimilarities for each variable. The overall dissimilarity index *I*_*diss*_ was then normalized to a 0–1 scale using the min-max normalization method. Finally, we calculated the *similarity index* by subtracting the normalized *I*_*diss*_ from 1. We analyzed the rice-suitable areas in both drainable and non-drainable zones, assuming a similarity index greater than 0.9.

We evaluated the potential wheat yield gains from improved soil drainage using an empirical model derived from field trials conducted at 16 experimental locations across the RFA region (see Supplementary Fig. 5a and Supplementary Table 3). The field trials compared wheat yield under traditional or no-drainage practices with yields obtained using improved drainage implemented using the broadbed maker (BBM). We fitted a linear regression model to quantify yield improvement as a function of waterlogging intensity (Supplementary Fig. 5b). This model was then applied to naturally drainable, waterlogging-prone grid cells (i.e., slope > 2%) to estimate the expected yield gains from implementing improved soil drainage practices^105^.

## Supplementary information

## Data Availability

The monthly climatology of agrohydrological variables and the annual climatology of waterlogging intensity [107] generated in this analysis are available on Zenodo at https://doi.org/10.5281/zenodo.17514460.

## Code availability

The code used for the agrohydrological simulations is publicly available on GitHub (https://github.com/mwakjira/Script-for-Ethio-AgHy-dataset-development-).
