# Supplementary material for: Agrohydrological analysis of waterlogging impacts and mitigation in Ethiopia
Source: NPJ Sustain Agric. 2026 Sep 2;4(1):74. doi: 10.1038/s44264-026-00179-0 (PMC13538058; doi:10.1038/s44264-026-00179-0)
Supplement: Supplementary file 1 [file 44264_2026_179_MOESM1_ESM.pdf]

Supplementary information

Agrohydrological analysis of waterlogging impacts and mitigation in Ethiopia

Mosisa Tujuba Wakjira[1*], Peter Molnar[2], Ruud Bartholomeus[3,4] , Teklu Erkossa[5] and Katrien Descheemaeker[1]

[1]Plant Production Systems Group, Wageningen University and Research, 6700AK Wageningen, The Netherlands

[2]Institute of Environmental Engineering, ETH Zurich, 8093 Zürich, Switzerland

[3]KWR Water Research Institute, 3433PE Nieuwegein, The Netherlands

[4]Soil Physics and Land Management (SLM), Wageningen University & Research (WUR), 6708PB Wageningen, The Netherlands

[5]Deutsche Gesellschaft für Internationale Zusammenarbeit (GIZ), Addis Ababa, Ethiopia

*Correspondence: Mosisa Tujuba Wakjira (email:mosisa.wakjira@wur.nl or mosisatujuba@gmail.com), Plant Production Systems Group, Wageningen University and Research, 6700AK Wageningen, The Netherlands

Supplementary Table 1: List of the 40 in situ surface runoff collection sites used for model calibration and evaluation. The data were collected from peer-reviewed and grey literature indicated under column reference. The reported methods of data creation (measurement and simulations) are indicated under column method.

Measurement site	Lat (° N)	Lon (° E)	Measurement periods	Method	Reference
Maybar	11.02	39.67	1982-1993	Runoff plot (30m x 6m)	[1] [2]
Hunde lafto	9.07	41.00	1983-1993	Runoff plot (30m x 6m)	
Andit Tid	9.80	39.72	1982-1992	Runoff plot (30m x 6m)	
Gununo	6.92	37.65	1985-1993	Runoff plot (30m x 6m)	
Anjeni	10.81	37.57	1985-1993	Runoff plot (30m x 6m)	
Dendi	9.13	37.12	2007-2009	Runoff plot (35m x 6m)	[3]
Holeta	9.07	38.48	2009-2010	Runoff plot (9m x 6m)	[4]
Debre Mawi	11.33	37.43	2008-2009	Runoff plot (30m x 6m)	[5]
Chefe donsa	8.96	39.11	2001-2002	Runoff plot (22m x 6m)	[6]
Dera	8.36	39.34	2004	Runoff plot (30m x 6m)	[7]
Yeku	12.52	39.07	2003	Runoff flow in channel	[8]
Guder	8.95	37.75	2003	Calibrated SWAT+ model	[9]
Dodota	11.50	39.92	1994-2000	Calibrated SWAT model	[10]
Keleta	8.12	39.46	1981, 1990	Calibrated SWAT model	[11]
Suluh	13.80	39.50	1992-2003	Calibrated HEC-HMS	[12]
Hare	6.25	37.55	1990-1999	Calibrated SWAT model	[13]
Bilate	7.50	37.94	1990-1999	Calibrated SWAT model	
Hagere Selam	13.65	39.17	2003-2004	Runoff plot (5m x 2m)	[14]
Adigudem	13.233	39.53	2005-2007	Runoff plot (19m x 5m)	[15]
Jigjiga	9.47	42.63	2004-2005	Runoff plot (22m x 1.8m)	[16]
Gum Selasa	13.25	39.53	2005-2007	Runoff plot (19m x 5m)	[17]
Maileba	13.23333	39.25	2007	Runoff plot (19m x 5m)	[17]
Melkasa	8.43	39.31	2004	Runoff plot (22m x 4m)	[18]
May zeg zeg	13.65	39.16667	2005-2010	Runoff plot (14m x 5m)	[19]
Giba	13.49	39.50333	2004-2009	Runoff plot (5.5m x 4m)	[20]
Haramaya	9.418	41.037	2000-2001	Runoff plot (6m x 4m)	[21]
Lenche Dima	11.8402	39.7321	2003-2004	Runoff plot (6m x 4m)	[22]
Mettu	8.334	35.537	1981-1998	NA (based on review)	[23]
Jeldu	9.371	37.975	1981-2010	Runoff plot (10m x 4m)	[24]
Gumera	11.7833	37.65	1981-2010	Runoff plot (20m x 5m)	[25]
Guder-ARS	11	36.9	1981-2010	Runoff plot (30m x 6m)	[25]
Kulumsa	8.0833	39.1717	1987-2010	GIS and CN methods	[26]
Kizin	11.733	37.65	1981-2010	Runoff flow in channel	[27]
Wonzima	11.74	37.675	1981-2010	Runoff flow in channel	[27]
Samet	10.116	39.879	1990-2013	Calibrated SWAT model	[28]
Shambu	9.568	37.077	1996-2010	Calibrated SWAT model	[29]
Ketar	7.681	39.27	1981-2010	Calibrated SWAT model	[30]
Daleti-Odagodere	10.25	35	1981-2010	Model (CN method)	[31]
Erer Gudda	9.4333	42.1267	1984-1995	Calibrated AnnAGNPS	[32]
Mieso	9.23	40.8	2003-2004	Runoff plot (2m x 2m)	[33]

Supplementary Table 2: List of the soil moisture measurements at 13 sites collected from published literature across the rainfed agricultural areas of Ethiopia

Experimental site	Lat (°N)	Lon (°E)	Years of measurement	Methods of measurement	Reference
Delanta	11.583	39.200	2005	Time Domain Reflectometer	[34]
Lenche Dima	11.840	39.731	2003-2004	Time Domain Reflectometer	[22]
Melkawoba	8.383	39.367	2005	Time Domain Reflectometer	[35]
Bishoftu	8.733	38.967	1986	Neutron probe and Gravimetric	[36]
Koga	11.300	37.150	2009	Gravimetric method	[37]
Chefe donsa	8.950	39.100	1999,2002	Gravimetric method	[38]
Ginchi	9	38	2002	Gravimetric method	[39]
Zequala	13.164	38.5	2009-2010	Gravimetric method	[40]
Abergelle	13.0285	38.9765	2010	Gravimetric method	[40]
Tero Jemjem	9.0180	38.219	1996-1999	Gravimetric method	[41]
Haramaya	9.3686	42.0127	1999	Time Domain Reflectometer	[42]
Melka Jebdu	9.610082	41.77584	2000	Gravimetric method	[43]
Bale mountains NP	6.75	39.75	2005	Gravimetric method	[44]

Supplementary Table 3: List of soil drainage evaluation field trial sites used to develop the empirical model (Figure 5 of the manuscript) for assessing the effectiveness of soil drainage in mitigating waterlogging across the rainfed agricultural areas of Ethiopia

Experimental site	Lat (°N)	Lon (°E)	Years of measurement	Reference
Akaki	8.867	38.784	1988, 1990	[45,46]
Bishoftu	8.693	39.055	1986-1988, 1990, 1998	[45–48]
Ginchi	9.018	38.219	2000-2001	[18]
Enewari	9.916	39.169	1986-1987	[45,47,49]
Dogolo	10.431	39.270	1986-1988	[45]
Dejen	10.209	38.133	1987, 1989	[45,49]
Wereilu	10.595	39.418	1986	[47]
Bahir Dar	11.550	37.340	2007	[49]
Bichena	10.444	38.188	1997	[49]
Delanta	11.583	39.200	2006	[50]
Chefe donsa	8.95	39.1	1997, 2000-2003	[51]
Kotu	9.531	39.466	1996-1997	[52]
Yilmana	11.5	37.333	2007	[53]
Sinana	10.281	39.161	2017-2018	[54]
Lume	8.586	39.170	2022	[55]
Sheno	9.354	39.302	1987	[47]

Supplementary Table 4: List of locations across the rainfed agricultural areas of Ethiopia, where the presence ("yes")/absence ("no") of waterlogging problems were reported. For waterlogging absence, location at which water conservation practices were studied were used as proxy.

Experimental site	Lat (°N)	Lon (°E)	Waterlogging presence	Reference
Ureal	7.684	36.827	Yes	[45]
Dipo	7.666	36.832	Yes	[45]
Bacho bore	7.653	36.849	Yes	[45]
Sinana	10.281	39.161	Yes	[46]
Ginchi	9.018	38.219	Yes	[18]
Debre Mawi	11.350	37.417	Yes	[47]
Girar Jarso	9.791	38.699	Yes	[48]
Enewari	9.916	39.169	Yes	[49–51]
Dejen	10.209	38.133	Yes	[49]
Bahir Dar	11.550	37.340	Yes	[49]
Merawi	11.420	37.140	Yes	[49]
Bichena	10.444	38.188	Yes	[49]
Dogolo	10.431	39.270	Yes	[49]
Becho	8.572	38.249	Yes	[52]
Lume	8.586	39.170	Yes	[52,53]
Chefe donsa	8.950	39.100	Yes	[6,54]
Akaki	8.867	38.784	Yes	[54]
Ambo	8.984	37.858	Yes	[54]
Bishoftu	8.693	39.055	Yes	[50]
Jemjem legebatu	9.060	38.120	Yes	[54]
Holeta	9.066	38.498	Yes	[54]
Mojo	8.795	39.267	Yes	[54]
Sheno	9.354	39.302	Yes	[55]
Tullu bolo	8.649	38.123	Yes	[54]
Woliso	8.847	38.810	Yes	[54]
Mehoni	12.798	39.627	Yes	[56]
Atsela	12.934	39.527	Yes	[56]
Wereilu	10.595	39.418	Yes	[51]
Delanta	11.583	39.200	Yes	[57]
Degem	9.839	38.594	Yes	[58]
Kotu-Angolela	9.531	39.466	Yes	[59]
Kuyu	9.78	38.44	Yes	[60]
Ensaro wayu	9.78	39.017	Yes	[61]
Enbese sar midir	10.834	38.305	Yes	[61]
Shebel berenta	10.485	38.161	Yes	[61]
Alamgena	8.8283	38.6086	Yes	[61]
Ilu	8.8319	38.2781	Yes	[61]
Gibmichu	8.8356	39.172	Yes	[61]
Dabat	12.9603	37.7429	Yes	[61]
Dessie	11.215	39.624	No	[61]
Albuko	10.774	39.550	No	[61]
Ambasel	11.755	39.238	No	[61]
Werebabo	11.454	39.931	No	[61]
West armachiho	13.499	36.471	No	[61]
Tefki	8.826	38.512	No	[61]
Ofla	12.553	39.408	No	[61]

Kito	7.662	36.793	No	45
Jiren	7.702	36.854	No	45
Unknown	7.275	35.556	Yes	62
Unknown	7.340	36.266	Yes	62
Unknown	7.667	36.047	Yes	62
Unknown	7.680	36.848	Yes	62
Unknown	7.732	37.739	Yes	62
Unknown	7.026	38.475	Yes	62
Unknown	8.752	37.506	Yes	62
Unknown	10.660	39.910	Yes	62
Unknown	10.909	37.261	Yes	62
Unknown	11.614	37.132	Yes	62
Unknown	11.954	37.649	Yes	62
Antsokia-Gemiza	10.723	39.780	No	63
Antsokia-Gemiza	10.667	39.784	No	63
Antsokia-Gemiza	10.609	39.799	No	63
Antsokia-Gemiza	10.545	39.835	No	63
Antsokia-Gemiza	10.473	39.859	No	63
Idaga Hamus	14.203	39.586	No	61
Hunde lafto	9.070	41.000	No	1 2
Dera	8.360	39.340	No	7
Yeku	12.520	39.070	No	8
Guder	8.954	37.750	No	9
Dodota	11.500	39.920	No	10
Keleta	8.120	39.460	No	11
Suluh	14.000	39.460	No	12
Adigudem	13.233	39.533	No	15
Maileba	13.233	39.250	No	17
Melkasa	8.430	39.310	No	18
May zeg zeg	13.650	39.167	No	19
Giba	13.490	39.503	No	20
Haramaya	9.369	42.013	No	21
Jeldu	9.371	37.975	No	23
Kulumsa watershed	8.083	39.172	No	25
Ketar (RV basin)	7.681	39.270	No	29
Daleti-Odagodere	10.250	35.000	No	30
Erer-Guddaa	9.433	42.127	No	31
Mieso	9.230	40.800	No	32

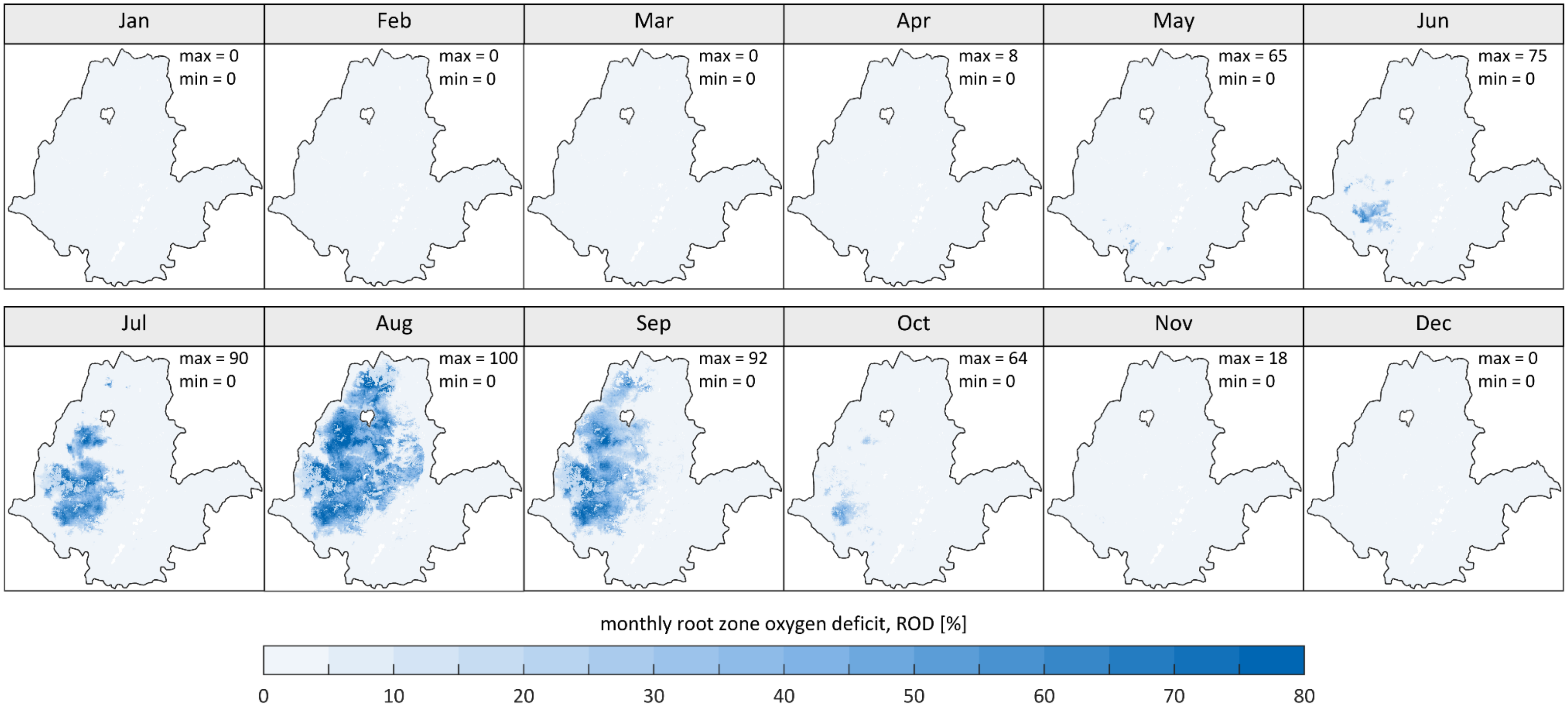

Supplementary Figure 1: Climatological mean monthly root zone oxygen deficit (ROD) across the rainfed agricultural region of Ethiopia, calculated for the reference period 1981-2010.

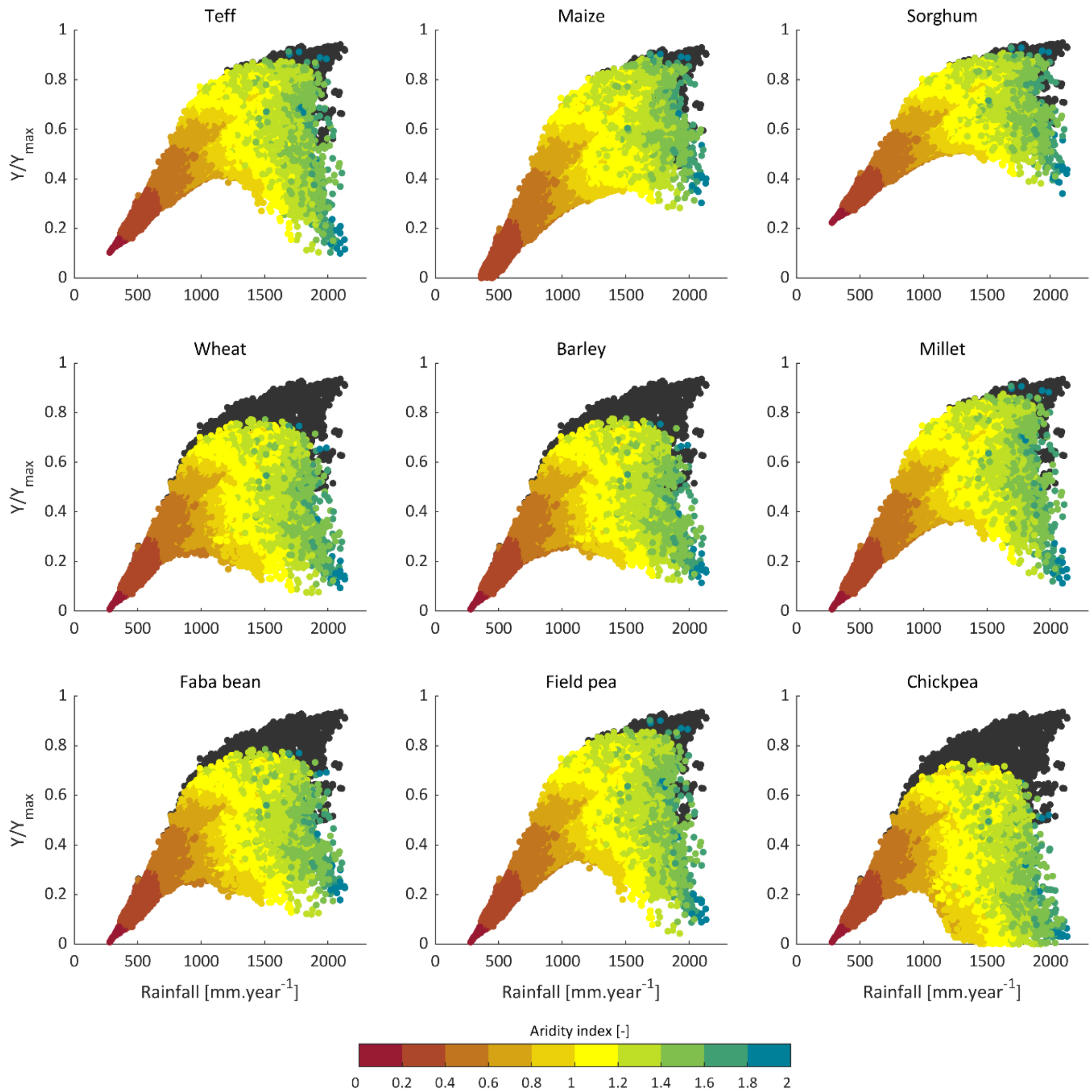

Supplementary Figure 2: Relative yield (Y/Y_{max}) of nine crops under different climatic regimes (represented by mean annual rainfall). Y/Y_{max} without waterlogging effect was computed using the FAO crop water productivity function [56]: 1-Ky(1-ETa/ETo), where Ky is crop-specific yield response factor obtained from [57], Y/Y_{max} with waterlogging effect was determined by subtracting the estimated waterlogging-induced yield loss from the non-waterlogged yield. The color gradient of the dots represents the aridity index of each grid cell. Each point corresponds to a 5 km x 5 km grid cell. Mean annual rainfall climatology (1981–2010) was obtained from CHIRPS.

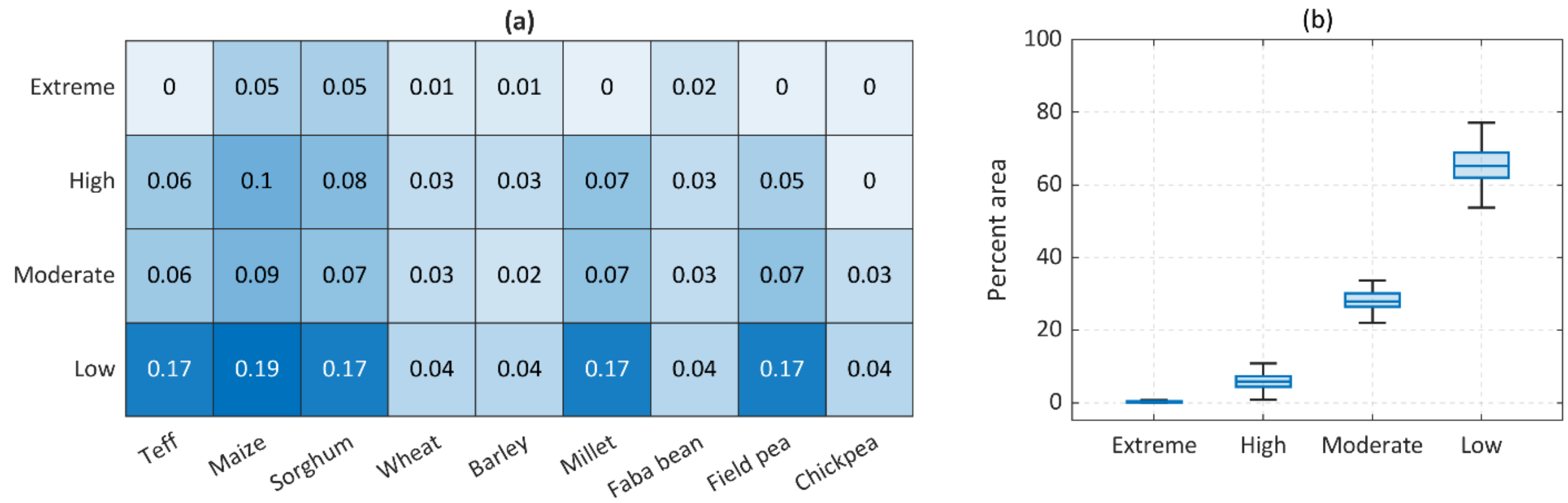

Supplementary Figure 3: Spatio-temporal variability of yield losses under waterlogging. a) Heatmap of coefficients of variation (CV) of annual crop yield, losses under extreme, high, moderate, and low waterlogging conditions across the RFA region of Ethiopia. The color gradients represent the CV values. b) Box charts showing the interannual variability in the proportion of waterlogging-prone areas (%) classified as experiencing extreme, high, moderate, and low waterlogging conditions across the RFA region of Ethiopia during the period 1981–2010.

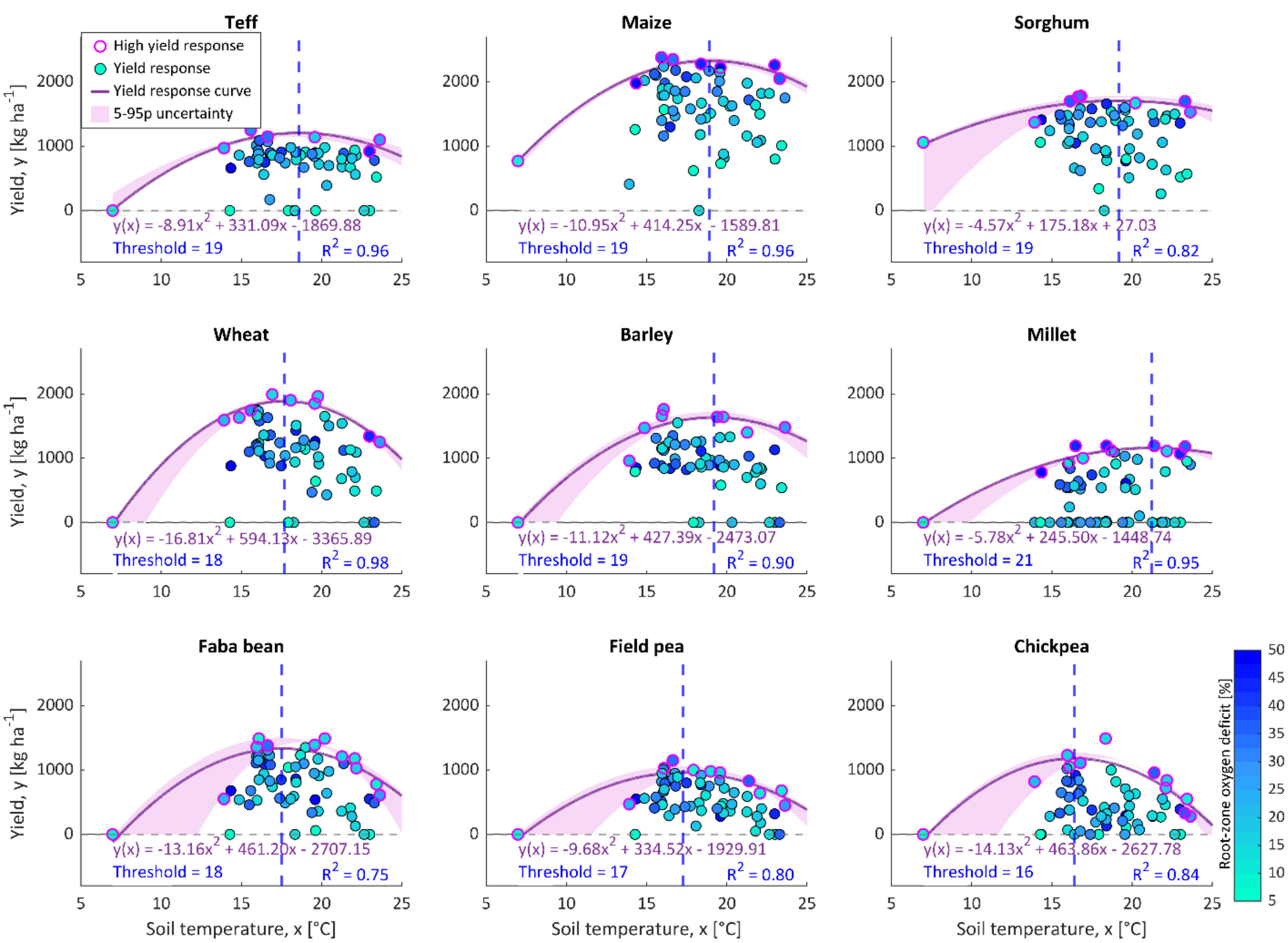

Supplementary Figure 4: Effect of soil temperature. Yield responses of nine crops to mean annual soil temperature across the rainfed agricultural (RFA) region of Ethiopia. Each data point represents the zone-averaged yield for an

administrative zone. The color gradient indicates average root-zone oxygen deficit. Pink circles represent the sampled high yield response (HYR) data used to define the model (purple line). The purple shade indicates the 5–95 percentile uncertainty band based on a leave-one-out uncertainty analysis. The blue dashed line marks the threshold soil temperature above which yield decreases.

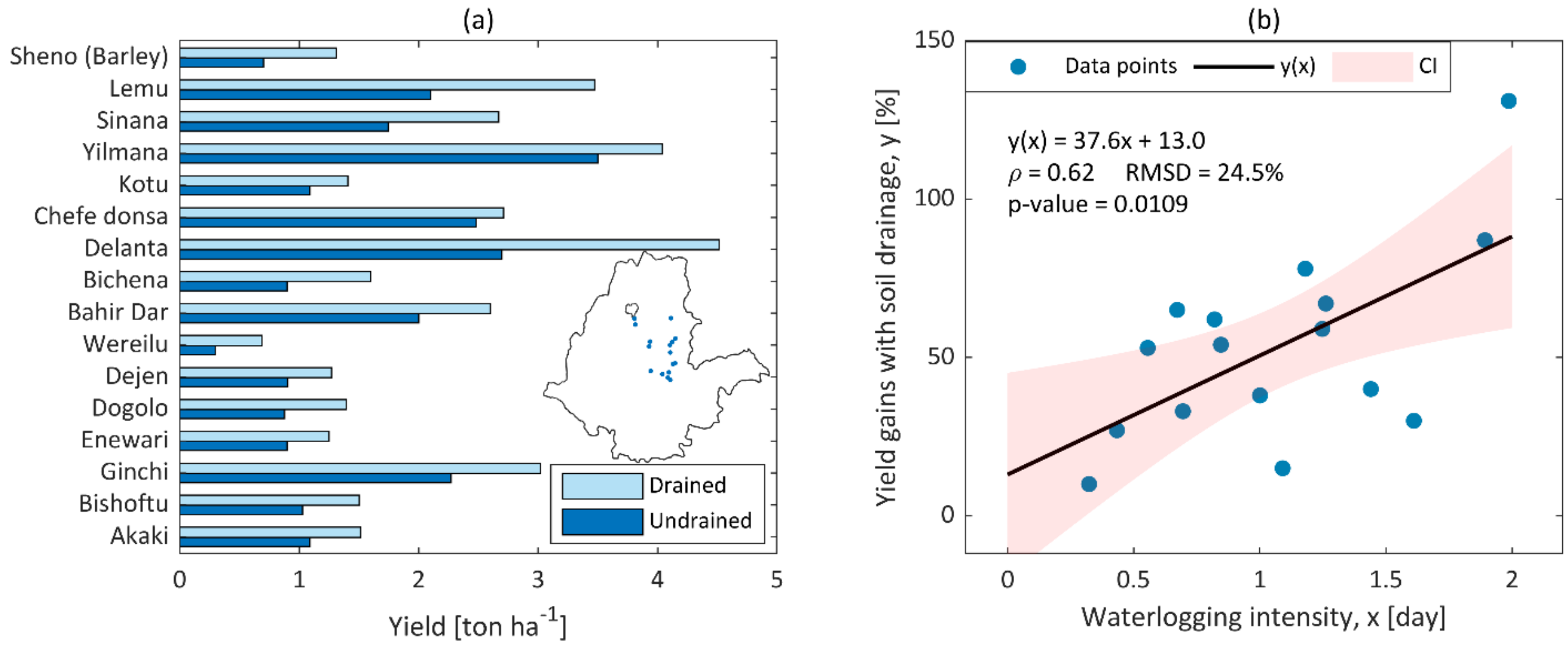

Supplementary Figure 5: Field trial data and regression model of yield gains under improved soil drainage. a) Comparison of wheat yield under well-drained and undrained vertisols at 16 field trial locations (shown in the inset map), based on published field trial data. b) linear regression model showing yield gains from improved soil drainage at the field trial sites under varying waterlogging intensity levels

References

1. Herweg, K. & Stillhardt, B. *The Variability of Soil Erosion in the Highlands of Ethiopia and Eritrea: Average and Extreme Erosion Patterns*. (1999).

2. Herweg, K. & Ludi, E. The performance of selected soil and water conservation measures - Case studies from Ethiopia and Eritrea. *Catena* **36**, 99–114 (1999).

3. Adimassu, Z., Mekonnen, K., Yirga, C. & Kessler, A. Effect of soil bunds on runoff, soil and nutrient losses, and crop yield in the central highlands of ethiopia. *L. Degrad. Dev.* **25**, 554–564 (2014).

4. Adimassu, Z., Alemu, G. & Tamene, L. Effects of tillage and crop residue management on runoff, soil loss and crop yield in the Humid Highlands of Ethiopia. *Agric. Syst.* **168**, 11–18 (2019).

5. Amare, T. *et al.* Combined effect of soil bund with biological soil and water conservation measures in the northwestern Ethiopian highlands. *Ecohydrol. Hydrobiol.* **14**, 192–199 (2014).

6. Erkossa, T., Stahr, K. & Gaiser, T. Effect of different methods of land preparation on runoff, soil and nutrient losses from a Vertisol in the Ethiopian highlands. *Soil Use

Manag. **21**, 253–259 (2006).

7. Welderufael, W. A., Le Roux, P. A. L. & Hensley, M. Quantifying rainfall-runoff relationships on the Dera Calcic Fluvic Regosol ecotope in Ethiopia. *Agric. Water Manag.* **95**, 1223–1232 (2008).

8. Collick, A. S. *et al.* A simple semi-distributed water balance model for the Ethiopian highlands. *Hydrol. Process.* **23**, 3718–3727 (2009).

9. Tumsa, B. C., Kenea, G. & Tola, B. The application of SWAT+ model to quantify the impacts of sensitive LULC changes on water balance in Guder catchment, Oromia, Ethiopia. *Heliyon* **8**, e12569 (2022).

10. Teso, E., Alamirew, T. & Olumana, M. Predicting Runoff Yield using SWAT Model and Evaluation of Boru Dodota Spate Irrigation Scheme , Arsi Zone , Southeastern Ethiopia. East Shoa Zone Irrigation Development Authority, Thesis, Adama, Ethiopia. 95–113 (2010).

11. Tibebe, D. & Bewket, W. Surface runoff and soil erosion estimation using the SWAT model in the Keleta Watershed, Ethiopia. *L. Degrad. Dev.* **22**, 551–564 (2011).

12. Abebe, B. A. Modeling the Effect of Climate and Land Use Change on the Water Resources in Northern Ethiopia: The Case of Suluh River Basin. *Department of earth secinces Institute of geographic scince, Freie University Berlin* (2014).

13. Wagesho, N., Jain, M. K. & Goel, N. K. Effect of Climate Change on Runoff Generation: Application to Rift Valley Lakes Basin of Ethiopia. *J. Hydrol. Eng.* **18**, 1048–1063 (2013).

14. Descheemaeker, K. *et al.* Runoff on slopes with restoring vegetation: A case study from the Tigray highlands, Ethiopia. *J. Hydrol.* **331**, 219–241 (2006).

15. Araya, T. *et al.* Effects of conservation agriculture on runoff, soil loss and crop yield under rainfed conditions in Tigray, Northern Ethiopia. *Soil Use Manag.* **27**, 404–414 (2011).

16. Welle, S., Chantawarangul, K. & Nontananandh, S. Effectiveness of Grass Strips as Barrier Against Runoff and Soil Loss in Jijiga Area. *Eff. Grass Strips as Barrier Against Runoff Soil Loss Jijiga Area* **558**, 549–558 (2006).

17. Gebreegziabher, T. *et al.* Contour furrows for in situ soil and water conservation, Tigray, Northern Ethiopia. *Soil Tillage Res.* **103**, 257–264 (2009).

18. Welderufael, W. A. & Woyessa, Y. E. Evaluation of surface water drainage systems for cropping in the Central Highlands of Ethiopia. *Agric. Water Manag.* **96**, 1667–1672 (2009).

19. Araya, T. *et al.* Medium-term effects of conservation agriculture based cropping systems for sustainable soil and water management and crop productivity in the Ethiopian highlands. *F. Crop. Res.* **132**, 53–62 (2012).

20. Araya, A. & Stroosnijder, L. Effects of tied ridges and mulch on barley (Hordeum vulgare) rainwater use efficiency and production in Northern Ethiopia. *Agric. Water Manag.* **97**,

841–847 (2010).

21. Woyessa, Y. E. & Bennie, A. T. P. Tillage-crop residue management and rainfall-runoff relationships for the alemaya catchment in eastern ethiopia. *South African J. Plant Soil* **24**, 8–15 (2007).

22. McHugh, O. V., Steenhuis, T. S., Berihun Abebe & Fernandes, E. C. M. Performance of in situ rainwater conservation tillage techniques on dry spell mitigation and erosion control in the drought-prone North Wello zone of the Ethiopian highlands. *Soil Tillage Res.* **97**, 19–36 (2007).

23. Bezuayehu, T., Ayele, G., Atnafe, Y., Jabbar, M. A. & Dubale, P. Nature and causes of land degradation in the Oromiya Region: A review. ILRI (International Livestock Research Institute), Nairobi, Kenya. *Socio-economics Policy Res. Work. Pap. 36* 82 (2002).

24. Jaleta, D., Mbilinyi, B. P., Mahoo, H. F. & Lemenih, M. Effect of Eucalyptus expansion on surface runoff in the central highlands of Ethiopia. *Ecol. Process.* **6**, 1–8 (2017).

25. Ebabu, K. *et al.* Land use, management and climate effects on runoff and soil loss responses in the highlands of Ethiopia. *J. Environ. Manage.* **326**, 116707 (2023).

26. Lindi, S., Hone, M., Eticha, B. & Nanesa, K. Estimation of Runoff Using CN Method and Arc GIS for Irrigation Water Accessibility at Kulumsa Watershed, Arsi Zone of Ethiopia. *Glob. Res. Environ. Sustain.* **2**, 1–15 (2024).

27. Monsieurs, E. *et al.* Impact of draining hilly lands on runoff and on-site erosion: A case study from humid Ethiopia. *Earth Surf. Process. Landforms* **41**, 513–525 (2016).

28. Beza, M., Hailu, H. & Teferi, G. Modeling and Assessing Surface Water Potential Using Combined SWAT Model and Spatial Proximity Regionalization Technique for Ungauged Subwatershed of Jewuha Watershed, Awash Basin, Ethiopia. *Adv. Civ. Eng.* **2023**, (2023).

29. Debela, S. K. & Feyessa, F. F. Rainfall-Runoff Modeling and Its Prioritization At Sub-Watershed Level Using Swat Model: a Case of Finca’Aa, Oromia, Western Ethiopia. *Water Conserv. Manag.* **6**, 22–29 (2022).

30. Sime, C. H., Demissie, T. A. & Tufa, F. G. Surface runoff modeling in Ketar watershed, Ethiopia. *J. Sediment. Environ.* **5**, 151–162 (2020).

31. Kassa, Y. Performance Assessment of Road Drainage Structures and Proposed Mitigation Measures: The case of Daleti-Odagodere Gravel Road in Benishangul-Gumuz Region. (Addis Ababa University, 2013).

32. Lindi, S. & Megersa, S. L. Prediction of Runoff and Sediment Yield Using AnnAGNPS Model: Case of Erer-Guda Catchment, East Hararghe, Ethiopia. **4**, (2014).

33. Welderufael, W. A. Quantifying rainfall-runoff relationships on selected benchmark ecotopes in Ethiopia: a primary step in water harvesting research. *Na* vol. NA (Wageningen University and Research, 2006).

34. Kebede, K. & Bekelle, E. Tillage Effect on Soil Moisture Storage and Wheat Yield on the

Vertisols of North Central Highlands of Ethiopia. *Ethiop. J. Environ. Stud. Manag.* **1**, 49–55 (2008).

35. Temesgen, M., Savenije, H. H. G., Rockström, J. & Hoogmoed, W. B. Assessment of strip tillage systems for maize production in semi-arid Ethiopia: Effects on grain yield, water balance and water productivity. *Phys. Chem. EARTH* **47**–**48**, 156–165 (2012).

36. Kamara, S. C. & Haque, I. *Kamara.Pdf*. (1989).

37. Chanie, T., Collick, A. S., Adgo, E., Lehmann, C. J. & Steenhuis, T. S. Eco-hydrological impacts of Eucalyptus in the semi humid Ethiopian Highlands: The Lake Tana Plain. *J. Hydrol. Hydromechanics* **61**, 21–29 (2013).

38. Teklu Erkossa. Tillage effects on physical qualities of a vertisol in the central highlands of Ethiopia. *African J. Environ. Sci. Technol.* **5**, 1008–1016 (2011).

39. Kidanu, S., Mamo, T. & Stroosnijder, L. Eucalyptus-wheat interaction on Ethiopian Nitosols. *Agric. Syst.* **80**, 151–170 (2004).

40. Zelelew, D. G., Ayimute, T. A. & Melesse, A. M. Evaluating the Response of In Situ Moisture Conservation Techniques in Different Rainfall Distributions and Soil-Type Conditions on Sorghum Production and Soil Moisture Characteristics in Drought-Prone Areas of Northern Ethiopia. *Water Conserv. Sci. Eng.* **3**, 157–167 (2018).

41. Taddese, G., Mohamed Saleem, M. A., Abyie, A. & Wagnew, A. Impact of grazing on plant species richness, plant biomass, plant attribute, and soil physical and hydrological properties of vertisol in East African highlands. *Environ. Manage.* **29**, 279–289 (2002).

42. Woldetsadik, K., Gertsson, U. & Ascard, J. Response of shallots to mulching and nitrogen fertilization. *HortScience* **38**, 217–221 (2003).

43. Abdelkdair, A. & Schultz, R. C. Water harvesting in a 'runoff-catchment' agroforestry system in the dry lands of Ethiopia. *Agrofor. Syst.* **63**, 291–298 (2005).

44. Yimer, F., Messing, I., Ledin, S. & Abdelkadir, A. Effects of different land use types on infiltration capacity in a catchment in the highlands of Ethiopia. *Soil Use Manag.* **24**, 344–349 (2008).

45. Mamo, T., Astatke, A., Srivastava, K. L. & Dibabe, A. *Improved Management of Vertisols for Sustainable Crop-Livestock Production in the Ethiopian Highlands : Synthesis Report 1986-92*. (1993).

46. Abebe, M., Mamo, T., Duffera, M. & Kidanu, S. Y. Crop Response to Improved Drainage of Vertisols in the Ethiopian Highlands. *J. Agron. Crop Sci.* **172**, 217–222 (1994).

47. Asamenew, G., Jutzi, S. C., Tedla, A. & J., M. Economic evaluation of improved vertisol drainage for food crop production in the Ethiopian Highlands. in *Management of Vertisols in sub-Saharan Africa* 263–283 (Addis Ababa, 1988).

48. Astatke, A., Jabbar, M., Saleem, M. A. M. & Erkossa, T. Technical and economic performance of animal-drawn implements for minimum tillage: Experience on vertisols

in Ethiopia. **38**, (2002).

49. Erkossa, T., Haileslassie, A. & MacAlister, C. Enhancing farming system water productivity through alternative land use and water management in vertisol areas of Ethiopian Blue Nile Basin (Abay). *Agric. Water Manag.* **132**, 120–128 (2014).

50. Mekonen, M., Tesfaye, K. & Bayu, W. Soil Drainage and Nutrient Management to Improve Productivity of Waterlogged Vertisols for Small-scale Farmers. *Eng. Int.* **1**, 80–92 (2013).

51. Erkossa, T., Menker, M. & Betrie, G. D. Effects of bed width and planting date on water productivity of wheat grown on vertisols in the Ethiopian Highlands. *Irrig. Drain.* **60**, 635–643 (2011).

52. Abera, T. *et al.* Research achievements in barley cultural practices in Ethiopia. in *Barley research and development in Ethiopia* 113–126 (2011).

53. Rutherford, A. S. *Broad Bed Maker Technology Package Innovations in Ethiopian Farming Systems: An*. (2008).

54. Lebay, M., Abiye, W., Taye, T. & Belay, S. Evaluation of Soil Drainage Methods for the Productivity of Waterlogged Vertisols in Jama District, Eastern Amhara Region, Ethiopia. *Int. J. Agron.* **2021**, (2021).

55. Fetene, S., Ahmed, M., Gonfa, A. & Amsalu, S. Participatory Demonstration of Durum Wheat Production Blending Vertisols Management Technologies in Lume District, East Shewa, Ethiopia. *Int. J. Agric. Econ.* **9**, 203–209 (2024).

56. Doorenbos, J. & Kassam, A. H. *Paper 33. Yield Response to Water*. *FAO Irrigation and Drainage Paper* (1979).

57. Allen, R. G., Pereira, L. S., Raes, D. & Smith, M. *Crop Evapotranspiration*. *Irrigation and Drainage Paper No. 56* (1998).
